# A Complete Skull of an Early Cretaceous Sauropod and the Evolution of Advanced Titanosaurians

**DOI:** 10.1371/journal.pone.0016663

**Published:** 2011-02-07

**Authors:** Hussam Zaher, Diego Pol, Alberto B. Carvalho, Paulo M. Nascimento, Claudio Riccomini, Peter Larson, Rubén Juarez-Valieri, Ricardo Pires-Domingues, Nelson Jorge da Silva, Diógenes de Almeida Campos

**Affiliations:** 1 Museu de Zoologia da Universidade de São Paulo, São Paulo, Brazil; 2 CONICET, Museo Paleontológico Egidio Feruglio, Trelew, Chubut, Argentina; 3 Instituto de Geociências, Universidade de São Paulo, São Paulo, Brazil; 4 Black Hills Institute of Geological Research, Hill City, South Dakota, United States of America; 5 University of Manchester, Manchester, United Kingdom; 6 Museo Patagónico de Ciencias Naturales, General Roca, Río Negro, Argentina; 7 Departamento de Biologia, Pontifícia Universidade Católica de Goiás, Goiânia, Goiás, Brazil; 8 Museu de Ciências da Terra, Departamento Nacional de Produção Mineral, Rio de Janeiro, Rio de Janeiro, Brazil; University of Chicago, United States of America

## Abstract

Advanced titanosaurian sauropods, such as nemegtosaurids and saltasaurids, were diverse and one of the most important groups of herbivores in the terrestrial biotas of the Late Cretaceous. However, little is known about their rise and diversification prior to the Late Cretaceous. Furthermore, the evolution of their highly-modified skull anatomy has been largely hindered by the scarcity of well-preserved cranial remains. A new sauropod dinosaur from the Early Cretaceous of Brazil represents the earliest advanced titanosaurian known to date, demonstrating that the initial diversification of advanced titanosaurians was well under way at least 30 million years before their known radiation in the latest Cretaceous. The new taxon also preserves the most complete skull among titanosaurians, further revealing that their low and elongated diplodocid-like skull morphology appeared much earlier than previously thought.

## Introduction

Titanosaurians are known as the most diverse group of sauropod dinosaurs, including one-third of all known genera of sauropods [1], [2]. Although their characteristic wide-gauge trackways have already been recorded in the Middle Jurassic [3], [4], titanosaurians are mostly known from Late Cretaceous postcranial remains [2]. Their abundance in Upper Cretaceous sediments has been regarded as a result of the successful radiation of a clade herein referred as the “advanced titanosaurians”. This radiation includes saltasaurids, nemegtosaurids and the closely related *Isisaurus* and *Diamantinasaurus* (but not the basal lithostrotian *Malawisaurus*), and was possibly triggered by the Cenomanian-Turonian global extinction of diplodocoid sauropods [5]–[8]. The presence of advanced titanosaurians in the Early Cretaceous, however, rests only on a few questionable fragmentary remains [9], [10]. The lack of an adequate sampling in the known fossil record has limited our understanding of the early evolution and initial diversification of advanced titanosaurians. Additionally, with the exception of a few well-preserved skulls from the Campanian and Maastrichtian of Madagascar and Asia [11], [12], little is known about the origin of their highly-modified skull anatomy, which shows remarkable convergences with diplodocids [8], [13], as illustrated by their posteriorly displaced nares, forward leaning quadrates, and narrow-crowned cylindrical teeth restricted to the front of the snout. Preservation of a nearly complete skull among sauropods is rare, probably because of their delicate construction. Our knowledge of titanosaurian skull anatomy, in particular, is mostly restricted to *Nemegtosaurus* [11] and *Rapetosaurus* [12], which are among the youngest records for this group. The absence of well-preserved titanosaurian cranial remains from the Early Cretaceous presents a major hurdle to understanding the fossil record of this group. Here we report on a new advanced titanosaurian sauropod discovered in outcrops of Aptian age in the Quiricó Formation of the Sanfranciscana Basin [14] (Figures S1, S2, and S3). Its discovery fills an important temporal gap and provides new information on the initial changes that led to the cranial anatomy of more derived titanosaurians. The present discovery also represents the first described titanosaurian skull for the South American continent.

## Methods

### Data matrix construction

The data matrix used in the phylogenetic analysis is based on a published phylogenetic analysis [15], with the addition of *Tapuiasaurus* and the recently published character scorings for three other Early Cretaceous titanosaurians (*Phuwiangosaurus*, *Tangvayosaurus*, *Diamantinasaurus*) [10], [16]. Nine skull characters were added to this dataset [2], [11], [13], resulting in a data matrix of 246 characters scored across 33 taxa (Text S2). Five of the multistate characters were treated as ordered, as in the original phylogenetic analysis [15].

### Heuristic tree search and support measures

The dataset was analyzed using equally weighted parsimony in TNT [17], [18] with a heuristic search of 1,000 replicates of Wagner trees followed by tree bisection-reconnection (TBR) branch swapping.

Two alternative support measures, Bremer support [19] and bootstrap resampling, were used to evaluate the robustness of the nodes of the most parsimonious trees. Bremer support [19] in TNT v.1.1 uses a combination of heuristic searches that save suboptimal trees with constraints for non-monophyly. One thousand bootstrap replicates were made using a heuristic tree search of 10 wagner trees (with random addition sequences) followed by TBR. Results of these replicates were summarized using absolute frequencies for each group.

### Nomenclatural Acts

The electronic version of this document does not represent a published work according to the International Code of Zoological Nomenclature (ICZN), and hence the nomenclatural acts contained in the electronic version are not available under that Code from the electronic edition. Therefore, a separate edition of this document was produced by a method that assures numerous identical and durable copies, and those copies were simultaneously obtainable (from the publication date noted on the first page of this article) for the purpose of providing a public and permanent scientific record, in accordance with Article 8.1 of the Code. The separate print-only edition is available on request from PLoS by sending a request to PLoS ONE, Public Library of Science, 1160 Battery Street, Suite 100, San Francisco, CA 94111, USA along with a check for $10 (to cover printing and postage) payable to “Public Library of Science”.

The online version of the article is archived and available from the following digital repositories: PubMedCentral (www.pubmedcentral.nih.gov/), and LOCKSS (http://www.lockss.org/lockss/). In addition, this published work and the nomenclatural acts it contains have been registered in ZooBank (http://www.zoobank.org/), the proposed online registration system for the ICZN. The ZooBank LSIDs (Life Science Identifiers) can be resolved and the associated information viewed through any standard web browser by appending the LSID to the prefix “http://zoobank.org/”. The LSID for this publication reads as follows: urn:lsid:zoobank.org:pub:C659B68A-0056-4BB8-A049-DCBC12BA9E06.

## Results

### Systematic Paleontology

Dinosauria Owen, 1842

Saurischia Seeley, 1888

Sauropoda Marsh, 1878

Titanosauria Bonaparte & Coria, 1993

*Tapuiasaurus macedoi* gen. et sp. nov.

urn:lsid:zoobank.org:act:9430DEDA-7C76-482B-8A23-912AC854836E.

urn:lsid:zoobank.org:act:39382702-C33C-4B92-AE49-AD93F696C052.

#### Etymology

From “*Tapuia*”, a generic name from the Jês indigenous language family used to designate tribes that inhabited the inner regions of Brazil, and *sauros*, Greek for lizard. The specific name honors Ubirajara Alves Macedo, who first discovered the deposits near Coração de Jesus.

#### Holotype

MZSP-PV (Museu de Zoologia da Universidade de São Paulo) 807, consists of an articulated partial skeleton composed of an almost complete skull and mandible, hyoid apparatus, atlas, axis, five cervical and five dorsal vertebrae and ribs, left sternal plate, right coracoid, right humerus, left radius, ulnae, metacarpals, femora, left fibula, and an almost complete left pes.

#### Horizon and locality

The skeleton was found in outcrops from the Quiricó Formation (Sanfranciscana Basin), at Embira-Branca Hills near Coração de Jesus City, in northern Minas Gerais, Brazil.

#### Age

The age of deposition in the Sanfranciscana Basin is well constrained by two magmatic events to the Lower Cretaceous. It postdates the eruption of the Paraná continental flood basalts dated at 138–128 Ma [20], and for the most part predates alkalic lavas and volcaniclastic rocks dated at 95–76 [21], which are intercalated with sandstones of the upper part of the basin fill. The lacustrine deposits of the Quiricó Formation, which are in the lower part of the Sanfranciscana sequence, are constrained to the Aptian based on the presence of sarcopterygian fishes [22], ostracods [23], and palynomorphs [24] (see Text S1 and Figures S1, S2, and S3).

#### Diagnosis

An advanced titanosaurian diagnosed by the following autapomorphies: hook-shaped posteroventral process of the quadratojugal; anterior process of the jugal tapering and forming most of the ventral margin of the antorbital fenestra; anterolateral tip of the pterygoid contacts the medial surface of the ectopterygoid. The new taxon is also diagnosed by the following unique combination of characters: deep fossa on the lateral surface of the maxilla between the antorbital fenestra and the subnarial foramen; elongated middle cervical vertebrae; posterior dorsal vertebrae with well-developed prespinal lamina and absence of hyposphene-hypantrum; deep fossae located below intraprezygapohyseal lamina; crescentric-shaped sternal plate; proximodistally long coracoid; elongated ulna and distally expanded radius.

### Description and comparisons

**Figure 1:**
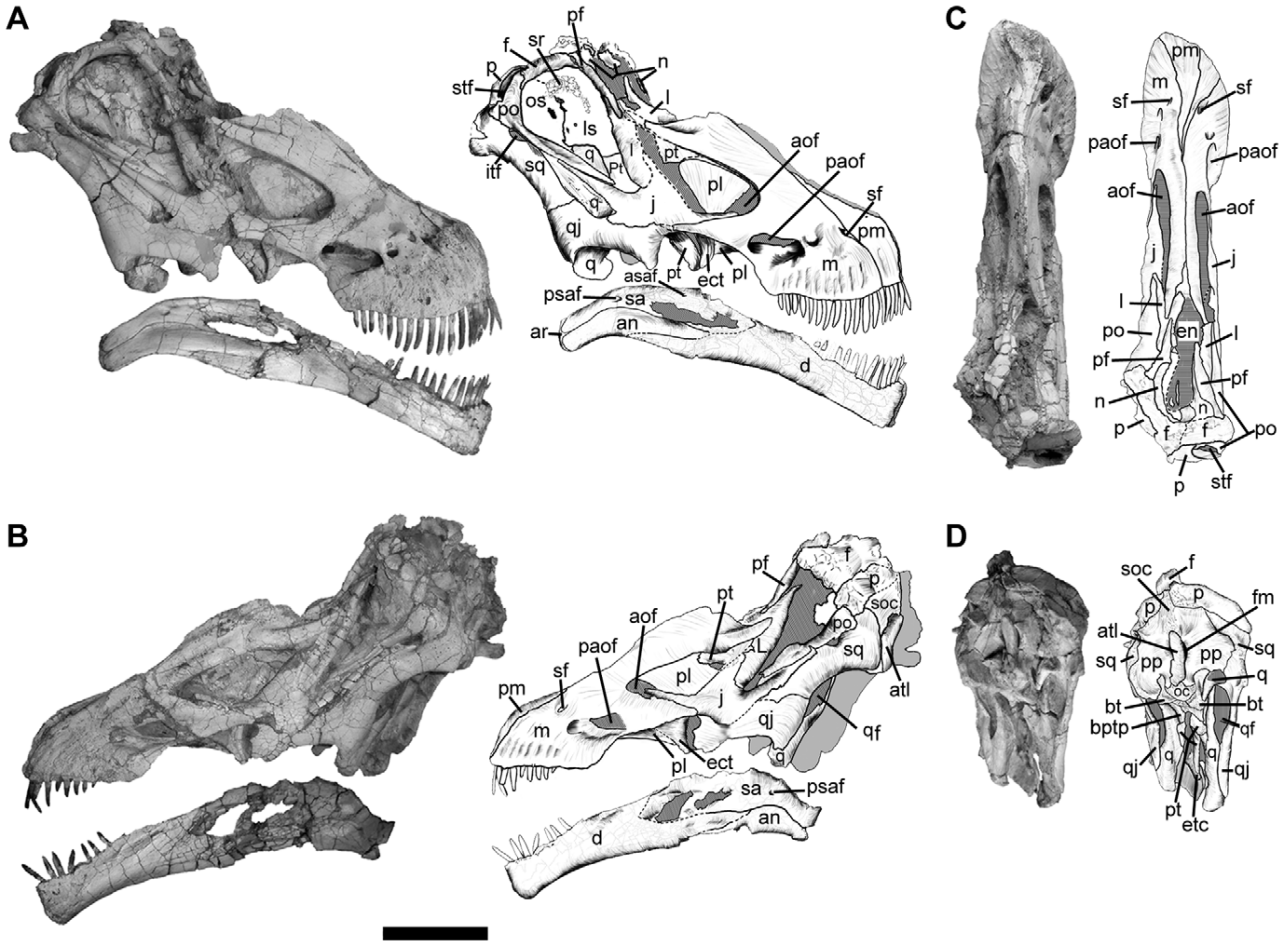
Skull of *Tapuiasaurus macedoi*, gen. n. sp. n. Photographs and half-tone drawings of the skull of the holotype MZSP-PV 807 in right lateral view (A), left lateral view (B), dorsal view (C), and occipital view (D). Abbreviations: *an*, angular; *aof*, antorbital fenestra; *ar*, articular; *asaf*, anterior surangular foramen; *atl*, atlas; *bt*, basal tubera; *bptp*, basipterygoid process; *d*, dentary; *ect*, ectopterygoid; *en*, external nares; *f*, frontal; *fm*, *forame magnum*; *itf*, infratemporal fenestra; *j*, jugal; *l*, lacrimal; *ls*, latrosphenoid; *m*, maxilla; *n*, nasal; *oc*, occipital condyle; *os*, orbitosphenoid; *p*, parietal; *paof*, preantorbital fenestra; *pf*, prefrontal; *pl*, palatine; *pm*, premaxilla; *po*, postorbital; *pp*, paroccipital process; *psaf*, posterior surangular foramen; *pt*, pterygoid; *q*, quadrate; *qf*, quadrate foramen; *qj*, quadratojugal; *sa*, surangular; *sf*, subnarial foramen; *soc*, supraoccipital; *sq*, squamosal; *sr*, sclerotic ring; *stf*, supratemporal fenestra. Scale bar represents 10 cm.

The skull of *Tapuiasaurus* (Figure 1), as in *Rapetosaurus* and *Nemegtosaurus*, has an elongated rostrum with narrow premaxillae that are not broadly exposed laterally, cylindrical teeth extending up to the level of the preantorbital fenestra, forward leaning quadrates, and external nares retracted to the level of the orbits [15], [25]. The antorbital fenestra is larger than in most macronarians (including *Nemegtosaurus*) but not as elongated as in *Rapetosaurus*. The premaxilla projects posterodorsally along the dorsal surface of the rostrum, as in *Nemegtosaurus* [11]. *Tapuiasaurus* also shares with *Rapetosaurus* and *Nemegtosaurus* an elongated post-dentigerous process of the maxilla. This process of the maxilla tapers posteriorly instead of forming a robust contact with the jugal, as in *Rapetosaurus*. A unique feature of *Tapuiasaurus* is the presence of a long anterior process of the jugal that covers the dorsal edge of the maxilla and forms most of the ventral margin of the antorbital fenestra. The lacrimal has a broad ventral process and a remarkably long anterior process on its dorsal extremity, a condition otherwise only known in *Rapetosaurus* [12]. The prefrontal has a short transverse articulation with the nasal and bears an anterior process as in nemegtosaurids [11], which is extremely long and thin as in *Rapetosaurus* [12].

As in most neosauropods the postorbital bears a posterior process and its jugal process is elongated and anteroposteriorly flattened. The postorbital of *Tapuiasaurus* contacts the parietal, excluding the frontal and squamosal from the supratemporal margin. The exclusion of the squamosal from the supratemporal fenestra also occurs in *Nemegtosaurus*, but contrasts with the more generalized condition of other titanosaurians in which the squamosal participates from the margins of this opening (including *Rapetosaurus*). The parietals have a broad surface separating the supratemporal fenestrae (as in other titanosaurids [15]). The occipital portion of the parietals and the supraoccipital are dorsoventrally low, as in *Nemegtosaurus* and *Rapetosaurus*. However, in contrast to these two taxa [15], the squamosal and postorbital of *Tapuiasaurus* are not ventrally shifted respect to the parietal so that the supratemporal fenestra is visible in lateral view. The squamosal participates on the margin of the supratemporal fenestra as in most titanosaurids, except for *Rapetosaurus* [12]. The quadrate projects anteroventrally and bears a deep fossa, but the lateromedial crushing of the specimen precludes determining if this was exposed posterolaterally as in nemegtosaurids. The quadratojugal bears an acute posteroventral process that directs (but fails to reach) the quadrate condyles, a feature that may have been present in *Rapetosaurus* given the articular facet for this bone preserved in the quadrate [12]. The anterior process of the quadratojugal expands ventrally so that the ventral margin of the quadratojugal is markedly concave as in *Nemegtosaurus*.

*Tapuiasaurus* also resembles nemegtosaurids in the presence of a reduced quadrate flange of the pterygoid [15] but bears a remarkably modified anterolateral process that contacts the medial surface of the ectopterygoid, as in *Diplodocus* [15]. The supraoccipital is low and resembles the condition of nemegtosaurids and some basal titanosaurians. *Tapuiasaurus* shares with advanced titanosaurians the presence of an acute non-articular ventral tip on the paroccipital process [15], [26]. The basal tubera are robust as in *Rapetosaurus* [12] and basal neosauropods. The basipterygoid processes are short, cylindrical shaped, and bear a sagittal ridge between them, closely resembling the condition in *Rapetosaurus* [25]. The basisphenoid contacts the medial surface of the quadrate and the quadrate flange of the pterygoid is reduced, as in nemegtosaurids [15].

The lower jaw of *Tapuiasaurus* (Figure 1) also shows derived features shared with *Nemegtosaurus* and *Rapetosaurus*, such as an unexpanded symphyseal region that is oriented perpendicular to the mandibular ramus and a smoothly curved tooth row in dorsal view. However, unlike *Nemegtosaurus*, the Meckelian groove does not reach the symphyseal region. The angular of *Tapuiasaurus*, however, is well exposed on the lateral surface of the posterior region of the mandible, distinguishing this taxon from the condition of most macronarians [15].

**Figure 2:**
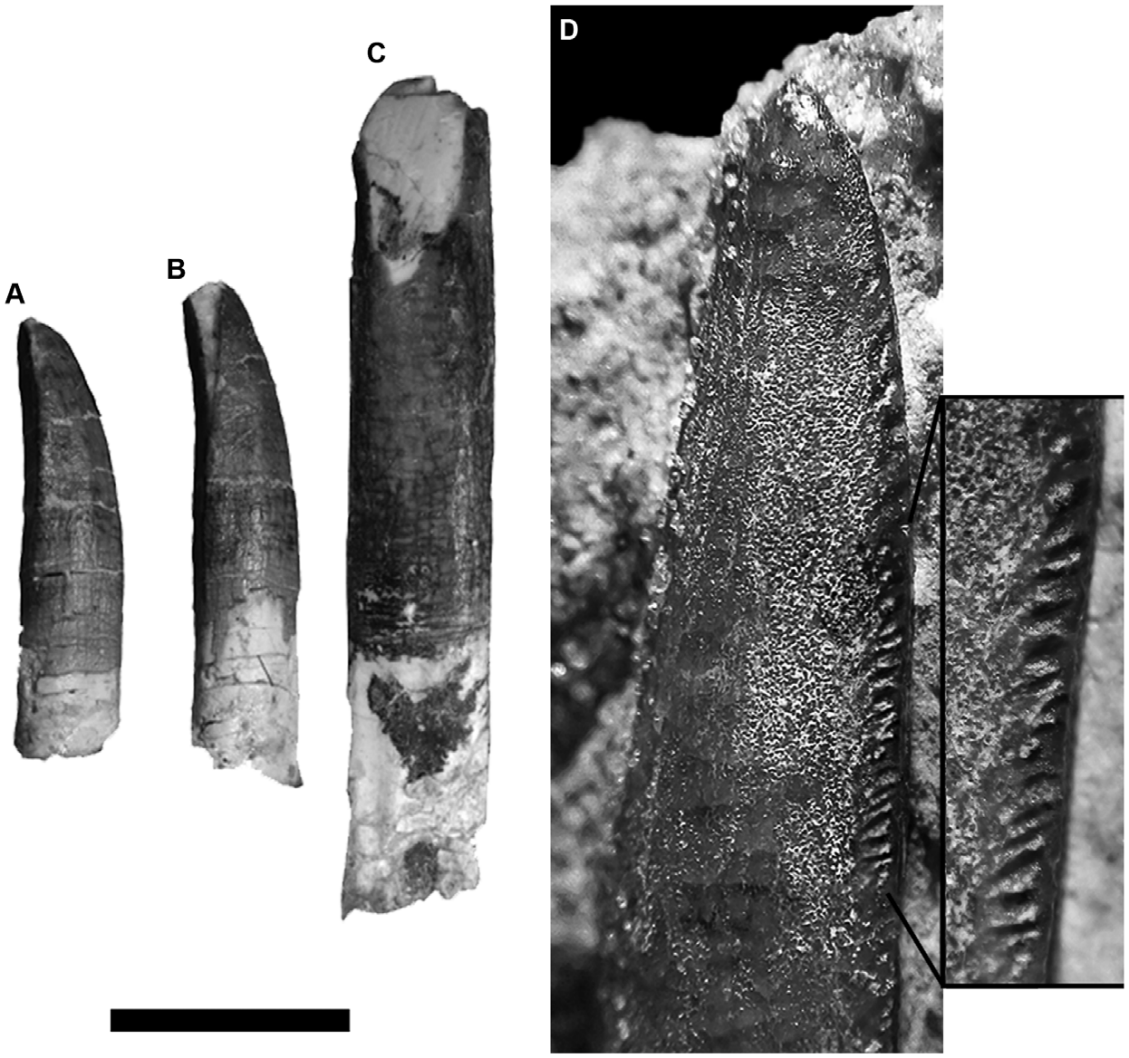
Dentition of *Tapuiasaurus macedoi*, gen. n. sp. n. Upper teeth in distal (A, B) and labial (C) views; premaxillary tooth in labial view (D) with detail of the premaxillary tooth showing the grooves along the carina. Scale bar represents 1 cm.

The toothrow extends up to the level of the preantorbital fenestra as in non-diplodocoid sauropods (Figure 1). All the upper and lower teeth of *Tapuiasaurus* are cylindrical and bear thin, regular carinae on their mesial and distal edges (Figure 2) that extend to the apex of each tooth. The enamel surface is slightly wrinkled, with diminute grooves that extend obliquely with respect to the apicobasal axis of the crown on the lingual and labial surfaces of the carinae. Older and more worn teeth tend to lack these grooves, presumably due to tooth-food abrasion.

The crowns of the upper tooth row are comparably broader mesiodistally than the crowns of the lower tooth row, a feature also present in *Nemegtosaurus* [11]. The upper teeth have slenderness index (SI; from [27]) values that range between SI 5.9 (in the 3^rd^ tooth) and SI 4.1 (in the 13^th^ tooth), whereas this index ranges between SI 4.9 (in the 2^nd^ tooth) and SI 3.4 (in the 9^th^ tooth) for the lower teeth. The upper teeth are also apicobasally longer than the lower teeth and the apicobasal length of the entire dentition decreases towards the posterior end of the tooth row. Up to three replacement teeth can be seen in the premaxilla and the maxilla.

The crowns of *Tapuiasaurus* bear both planar high-angled and V-shaped wear facets on upper and lower teeth (Figure 2). The V-shaped wear facets are labiolingually narrow and only slightly developed and they only occur in a few teeth. The high-angled wear facets are much more extensive and are present in most teeth of the anterior region of the toothrow. Given the high-angled wear facets are more extensive and occur in highly worn crowns they probably occur in a final stage of the ontogeny of the teeth. The unusual combination of high-angled and V-shaped wear facets has also been described for *Nemegtosaurus* [11], whereas most other sauropods have wear facets of either one type or the other.

**Figure 3:**
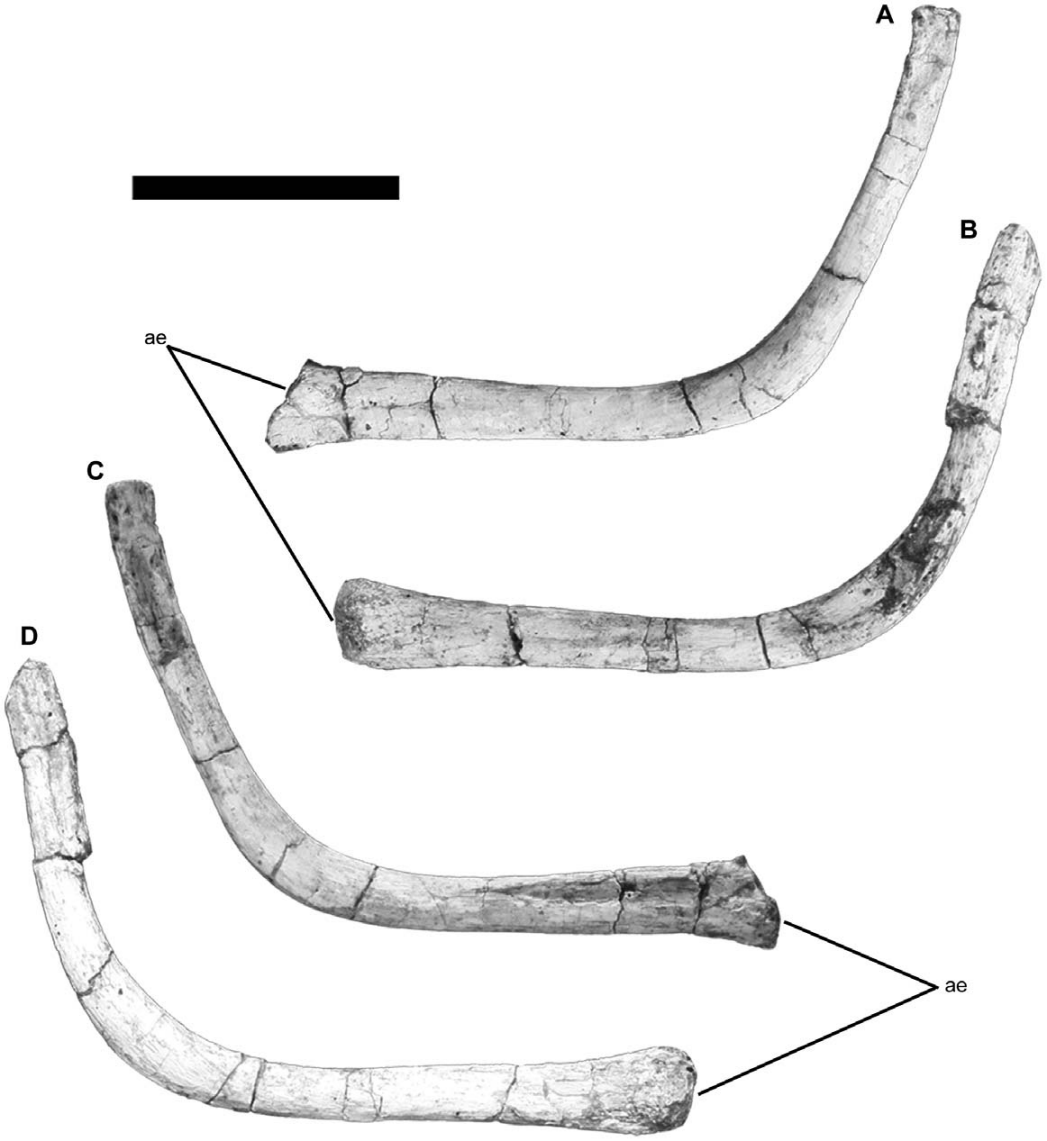
Hyoid apparatus of *Tapuiasaurus macedoi*, gen. n. sp. n. Left element in lateral view (A); right element in medial view (B); left element in medial view (C); right element in lateral view (D). Abbreviations: *ae*, anterior end. Scale bar represents 5 cm.

The hyoids are two long and curved, boomerang-like bones (Figure 3) that were preserved in place. They are disposed parallel to each other and in a posteroventral position in respect to the posterior end of the mandible and squamosal. The wide concave side of each hyoid faces towards the skull. The thicker and more porous anterior end of each element is at the level of the articular. Both hyoids have an approximate length of 17.5 cm (right with 17.6 cm and left with 17.3 cm). The anterior half of each hyoid (right with 9.4 cm and left with 8.6 cm) extends posteriorly as a rod-like element. At that level, the posterior half of the hyoid's body curves dorsally in 120 degrees towards the posterior end of the squamosal. The dorsally extended posterior halves of both right and left hyoids are 8.2 cm and 8.7 cm long, respectively, and are diagenetically compressed laterally.

**Figure 4:**
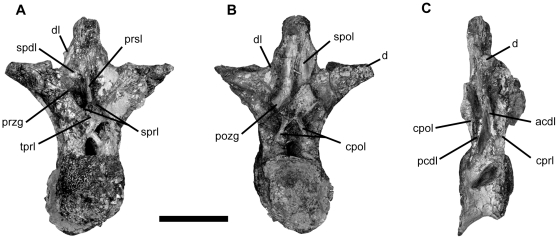
Vertebrae of *Tapuiasaurus macedoi*, gen. n. sp. n. Dorsal vertebra of the holotype MZSP-PV 807 in anterior (A), posterior (B), and right lateral (C) views. The lamination nomenclature follows previous workers [28], [33]. Abbreviations: *acdl*, anterior centrodiapophyseal lamina; *cpol*, centropostzygapophyseal lamina; *cprl*, centroprezygapophyseal lamina; *d*, diapophysis; *dl*, diapophyseal lamina; *pcdl*, posterior centrodiapophyseal lamina; *pozg*, postzygapophysis; *prsl*, prespinal lamina; *przg*, prezygapophysis; *spdl*, spinodiapophyseal lamina; *spol*, spinopostzygapophyseal lamina; *sprl*, spinoprezygapophyseal lamina; *tprl*, intraprezygapophyseal lamina. Scale bars represents 10 cm.

Presacral vertebrae are opisthocoelous and highly pneumatized, with camellate internal structure and large pleurocoels, as in other titanosauriforms (Figure 4). The mid-cervical centra are more than four times as long as high, as in non-saltasaurid titanosaurians [15]. Mid-dorsal vertebrae have a large diapophyseal lamina [28] that meet the spinopostzygapophyseal laminae along the neural spine, and share with advanced titanosaurians an extensive prespinal lamina and the absence of a hyposphene-hypantrum [2], [15], [28], [29]. The dorsal ribs are plank-like with a large proximal pneumatopore as in titanosauriforms.

**Figure 5:**
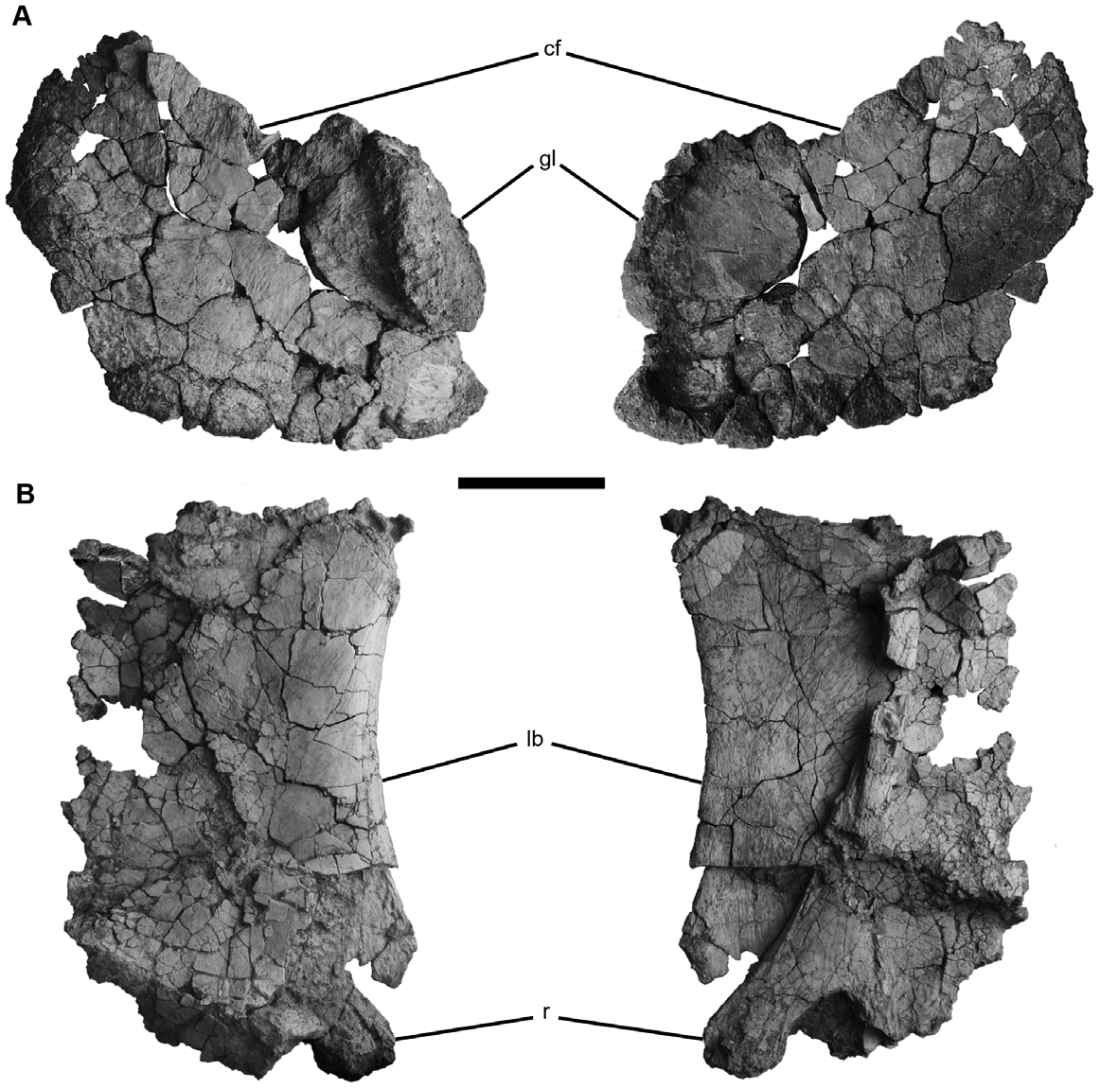
Coracoid and sternal plate of *Tapuiasaurus macedoi*, gen. n. sp. n. Right coracoid (A) in lateral (left) and medial (right) views; Right sternal plate (B) in anterior (left) and posterior (right) views. Abbreviations: c*f*, coracoid foramen; *gl*, glenoid fossa; *lb*, lateral border. Scale bar represents 10 cm.

**Figure 6:**
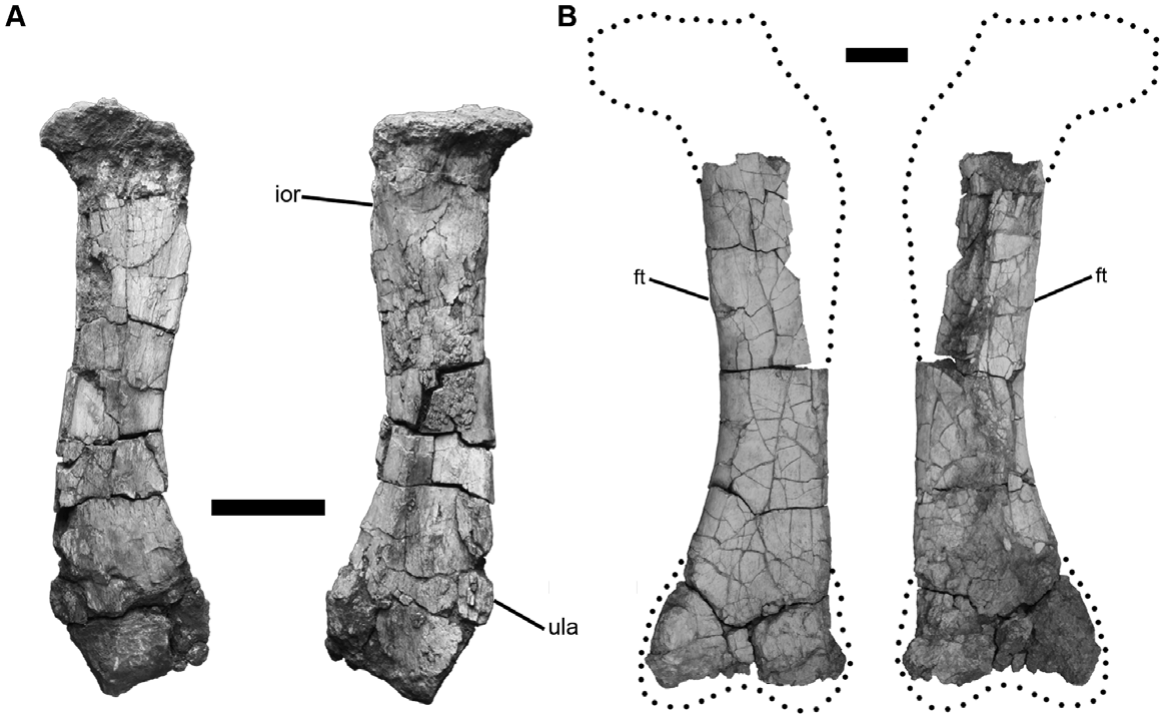
Radius and femur of *Tapuiasaurus macedoi*, gen. n. sp. n. Left Radius (A) in anterior (left) and posterior (right) views; Left femur (B) in anterior (left) and posterior (right) views. Abreviations: *ft*, fourth trochanter; *ior*, interosseous ridge for attachment of interosseous membrane; *ula*, ulnar articular facet. Scale bar represents 10 cm.

The coracoid is proximodistally long (Figure 5), and the distal end of the radius is expanded as in *Rapetosaurus* and saltasaurids [15], but differs from the derived condition of the latter group in being anteroventrally rounded. Titanosaurian characters present in the appendicular skeleton include a crescentric-shaped sternal plate (Figure 5) and a well-developed olecranon process on the ulna [15]. *Tapuiasaurus* shares with *Rapetosaurus* and saltasaurids an expanded distal end of the radius, but lacks the robust ulnar proportions of saltasaurids (Figure 6). The fragmentary hindlimb elements have a combination of characters supporting titanosaurian affinities such as lateromedially broad femoral shaft (Figure 6), broad pedal phalanges, laterally deflected unguals, and ungual I and II subequal in size.

### Phylogenetic analysis

**Figure 7:**
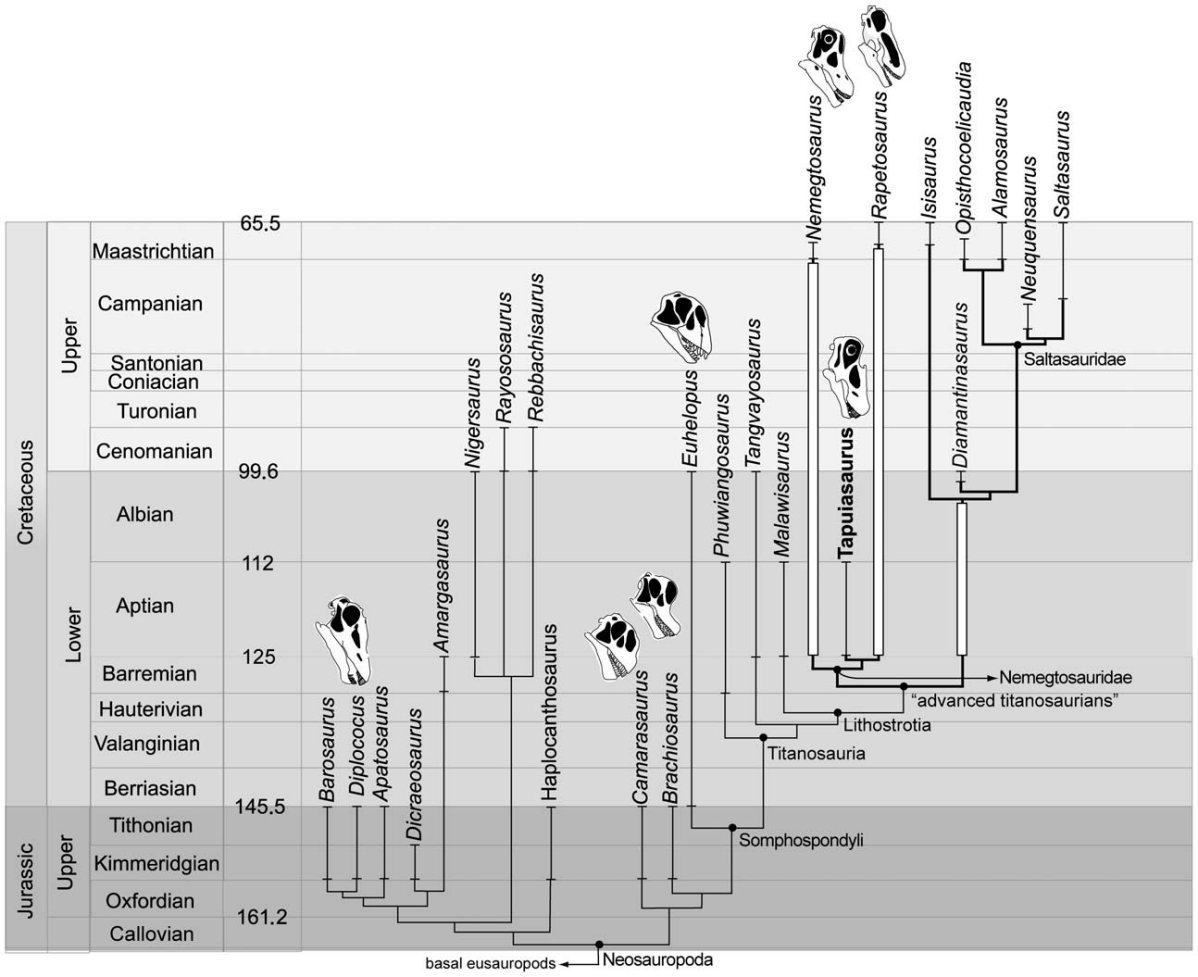
Calibrated phylogeny of Neosauropoda. Summarized strict consensus tree showing the relationships among neosauropod dinosaurs and the phylogenetic position of *Tapuiasaurus macedoi* gen. n. sp. n. The support values (Bremer/Bootstrap) for the nodes labelled in the figure are: Neosauropoda (1/-), Somphospondyli (2/68), Titanosauria (2/58), Lithostrotia (3/68), “advanced titanosaurians” (3/65), Nemegtosauridae (1/-), Saltasauridae (1/-). See Figure S4 for a complete strict consensus tree including all sauropod terminal taxa used in the analysis. Age of first appearance for taxa used in the calibrated phylogeny are given in Text S4.

Two most parsimonious trees of 445 steps (CI = 0.613, RI = 0.789) were found in all replicates, using the collapsing rule 3 for zero-length branches [30], the strict consensus of which is shown in Figure 7 (see also Figure S4). Bremer and bootstrap support values for the nodes of the consensus tree are given in Figure 7 for selected nodes (see Figure S5 for other support values and Figure S6 for support values on the reduced consensus tree). A list of unambiguous synapomorphies supporting the nodes of the strict consensus of Figure S4 is shown in Text S3.

The branch support values for several nodes within Lithostrotia are moderate to low, with Bremer support values ranging between 1 and 3 and bootstrap frequencies ranging between 55 and 76 (Figure S5). These relatively low values are caused because several incomplete taxa (i.e., *Diamatinasaurus*, *Isisaurus*, and *Nemegtosaurus*; see results below) can be placed in alternative positions without producing a marked increase in tree length [31]. In order to evaluate this issue, several runs were performed to test the degree of character support for positioning *Tapuiasaurus* deeply nested within advanced titanosaurians.

#### Constrained searches

Using constrained searches in TNT to find the most parsimonious trees that depict selected taxa in alternative positions (using the *force* command before the heuristic tree searches), we found that the three most feebly supported taxa within Lithostrotia are the fragmentary Early Cretaceous *Diamantinasaurus*, the latest Cretaceous *Isisaurus*, and *Nemegtosaurus* (which lacks postcranial elements). These taxa can be placed in alternative positions within Lithostrotia (including at the base of this clade), increasing the tree in only one to three steps.

However, placing *Tapuiasaurus* at the base of Lithostrotia requires six extra steps if it is forced to be only in a slightly more basal position, but more derived than *Malawisaurus* (i.e., as the sister group of the node of advanced titanosaurians, see Figure 7). Several derived characters of the skull and mandibles of *Tapuiasaurus* are responsible for this marked increase in tree length. In particular, the derived characters shared by *Tapuiasaurus* and *Rapetosaurus* that are absent in *Nemegtosaurus* are the ones that increase the tree length if *Tapuiasaurus* is placed more basally in the tree. These include the presence of an antorbital fenestra that is subequal or larger than the maximum orbital diameter (character 6), posteriorly tapering jugal process of the maxilla (character 235), narrow and elongated prefrontals (character 239), presence of a sagittal ridge between the basipterygoid processes (character 242), robust basal tubera (character 48), and unexpanded dentary at the mandibular symphysis (character 55).

Furthermore, placing *Tapuiasaurus* in even more basal positions within Titanosauria requires an increasingly higher number of extra steps: 7 steps if forced basal to *Malawisaurus*, 9 steps if forced basal to *Tangvayosaurus*, and 10 steps if forced basal to *Phuwiangosaurus*. Similar increase in tree length is obtained if most of the other derived lithostrotians are forced to be in a more basal position within Titanosauria (i.e., *Alamosaurus* [7 extra steps], *Rapetosaurus* [7 extra steps], *Saltasaurus* [9 extra steps], *Opisthocoelicaudia* [10 extra steps], *Neuquensaurus* [10 extra steps]). Therefore, based on the context of this dataset, the derived position of *Tapuiasaurus* can be considered as robust as that of other derived lithostrotians. Such a derived position of *Tapuiasaurus*, despite being well supported by character data, creates three extensive ghost lineages that are critical to understand the pace of diversification of advanced titanosaurians (see below).

#### Reduced consensus for Bremer support and Bootstrap/Jackknife analyses

Reduced consensus can be used to reveal common phylogenetic information ignoring the position of unstable taxa. This is usually done in the set of most parsimonious trees. However, it can be applied to other sets of topologies, such as the suboptimal trees found during a Bremer analysis or to the set of trees found during bootstrap/jackniffe pseudoreplicates (as implemented in TNT). In this way, support values can be calculated for a subset of the taxa present in the data matrix, ignoring the effect that highly unstable or incomplete taxa can have in the support measures [31]. The result of this type of analysis shows higher character support for the inclusion of *Tapuiasaurus* among advanced titanosaurians (Figure S6), with Bremer values of up to 3 and bootstrap values of up to 83, mainly caused by the large number of derived features shared by the skulls of *Tapuiasaurus* and *Rapetosaurus*.

## Discussion

The phylogenetic analysis shows that *Tapuiasaurus* is deeply nested within an advanced titanosaurian clade formed by nemegtosaurids and saltasaurids (as well as *Isisaurus* and *Diamantinasaurus*), as the sister taxon of *Rapetosaurus* (Figure 7; Figure S4). The affinities between these two genera are supported by several cranial features that are absent in *Nemegtosaurus*, including the length of the antorbital fenestra subequal or larger than the orbit, robust basal tubera, unexpanded mandibular symphysis, maxillary jugal process tapering posteriorly, and prefrontal with narrow and elongated anterior process. The advanced titanosaurian clade is supported by the presence of postcranial features such as a proximodistally elongated coracoid, procoelous middle and posterior caudal vertebrae, and distal condyles expanded on the anterior surface of the humerus [15], only the first one being preserved in *Tapuiasaurus*. The placement of *Tapuiasaurus* among advanced titanosaurians is however robustly supported, given that its placement as the sister group of that clade requires seven extra steps in the parsimony analysis. This result also holds for other advanced titanosaurians, suggesting that the derived position of *Tapuiasaurus* can be considered as robust as that of other taxa belonging to that clade [Figures S5 and S6].

The derived position of *Tapuiasaurus* and its Aptian age reveal the existence of multiple and extensive ghost lineages [5], which lengthen to 30 million years the diversification of advanced titanosaurians (Figure 7). Early Aptian diversification of advanced titanosaurians may explain their global distribution on landmasses that were comparatively isolated by the Late Cretaceous [32].

The complete skull of *Tapuiasaurus* indicates that the basic cranial morphology of advanced titanosaurians (narrow crowns, elongate rostrum, retracted naris, and an anteroventrally inclined quadrate), previously known only in the latest Cretaceous *Rapetosaurus* and *Nemegtosaurus*, was acquired at the initial diversification of the group during the Early Cretaceous. Furthermore, the discovery of *Tapuiasaurus* in Aptian rocks of South America demonstrates that these advanced titanosaurians with a derived skull morphology coexisted with other lineages of large herbivores, such as the more plesiomorphic broad-crowned titanosauriforms and diplodocoid sauropods, during a period of major changes in terrestrial ecosystems that involved the diversification of flowering plants and appearance of several modern lineages of vertebrates [6]. The long period of coexistence of these sauropod lineages suggests that the evolutionary success of advanced titanosaurians after the Cenomanian-Turonian extinction is better explained by an opportunistic radiation rather than by competitive replacement.

## Supporting Information

Figure S1 **Location of the Sanfranciscana basin.** Location and geological sketch-map of the basin showing the local of occurrence of *Tapuiasaurus macedoi* gen. n. sp. n.
(TIF)

Figure S2 **Stratigraphy of the Sanfranciscana basin.**
(TIF)

Figure S3 **Columnar section and plan view of occurrence of fossil bones of** ***Tapuiasaurus macedoi*** **gen. n. sp. n.**
(TIF)

Figure S4 **Strict consensus of the two most parsimonious trees found in the phylogenetic analysis.**
(TIF)

Figure S5 **Bremer and bootstrap support values for the nodes of the consensus tree.**
(TIF)

Figure S6 **Bremer and bootstrap support values for the nodes on a reduced consensus tree.** The tree shows support values that result from applying reduced consensus during the Bremer and Bootstrap analyses, ignoring the alternative positions of the most unstable advanced titanosaurians included in the analysis (i.e., *Nemegtosaurus, Diamantinasaurus*, and *Isisaurus*).
(TIF)

Text S1 **Geological Setting.**
(DOC)

Text S2 **Character list and data matrix used in phylogenetic analysis.** Character definitions 1 to 234 are from [15] and have the same numeration as in the original publication. The additional characters are either new or taken from [2] and their respective sources are cited along with the character number of the original publication. Characters 8, 37, 64, 66, and 198 were set as ordered. The data matrix corresponds to the phylogenetic analysis published in [15] with the following modifications. Character scorings for *Euhelopus* were taken from the corrected list provided in [34]. Character scorings for *Phuwiangosaurus* and *Tangvayosaurus* were taken from [16] and those of *Diamantinasaurus* follow those given by [10].
(DOC)

Text S3 **List of unambiguous synapomorphies.**
(DOC)

Text S4 **Age of first appearance for taxa used in the calibrated phylogeny.**
(DOC)
