# Supplementary figures and images for: A Complete Skull of an Early Cretaceous Sauropod and the Evolution of Advanced Titanosaurians
Source: PLoS One. 2011 Feb 7;6(2):e16663. doi: 10.1371/journal.pone.0016663 (PMC3034730; doi:10.1371/journal.pone.0016663)

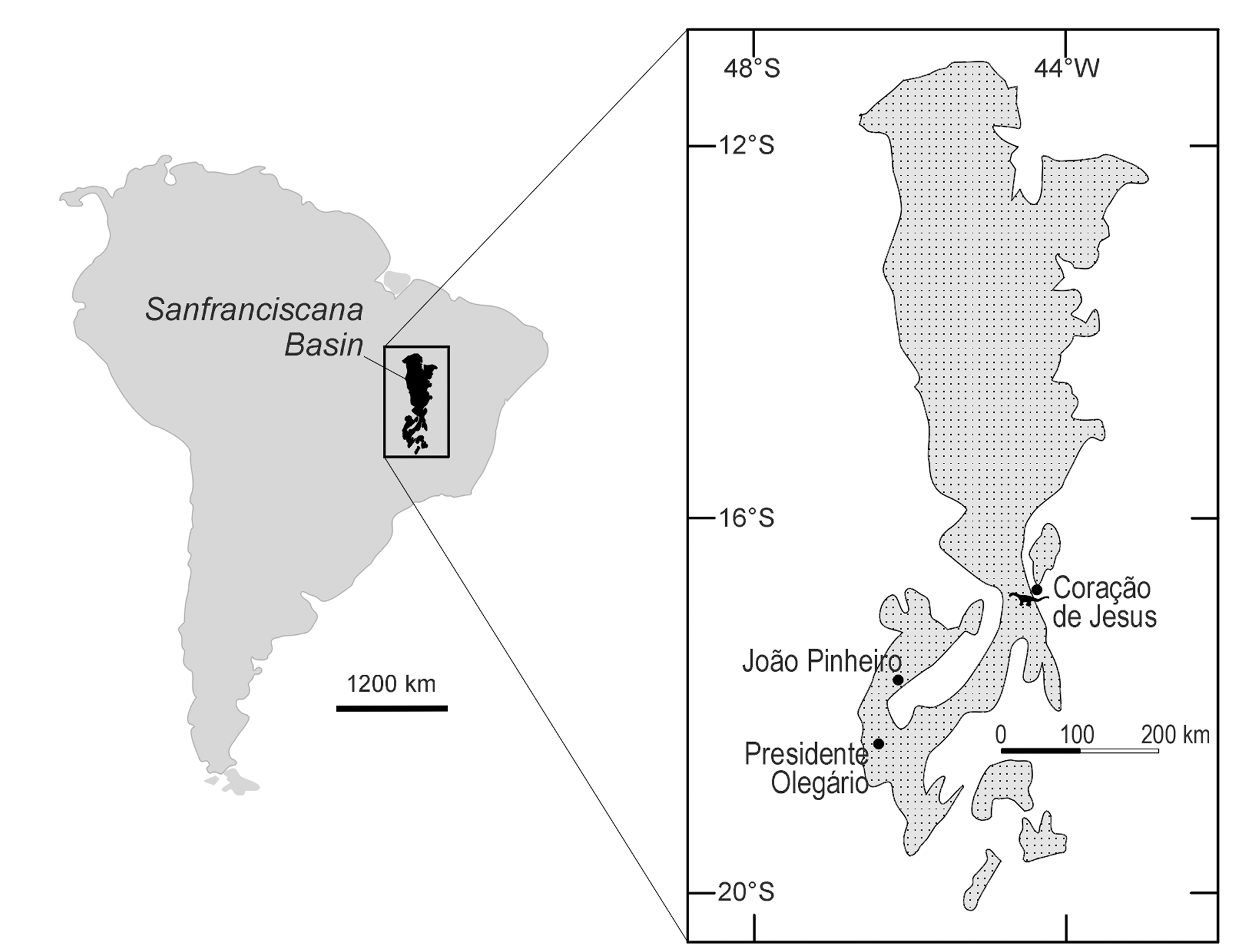

Supplement: Figure S1 — Location of the Sanfranciscana basin. Location and geological sketch-map of the basin showing the local of occurrence of Tapuiasaurus macedoi gen. n. sp. n. (TIF) [file pone.0016663.s001.tif]

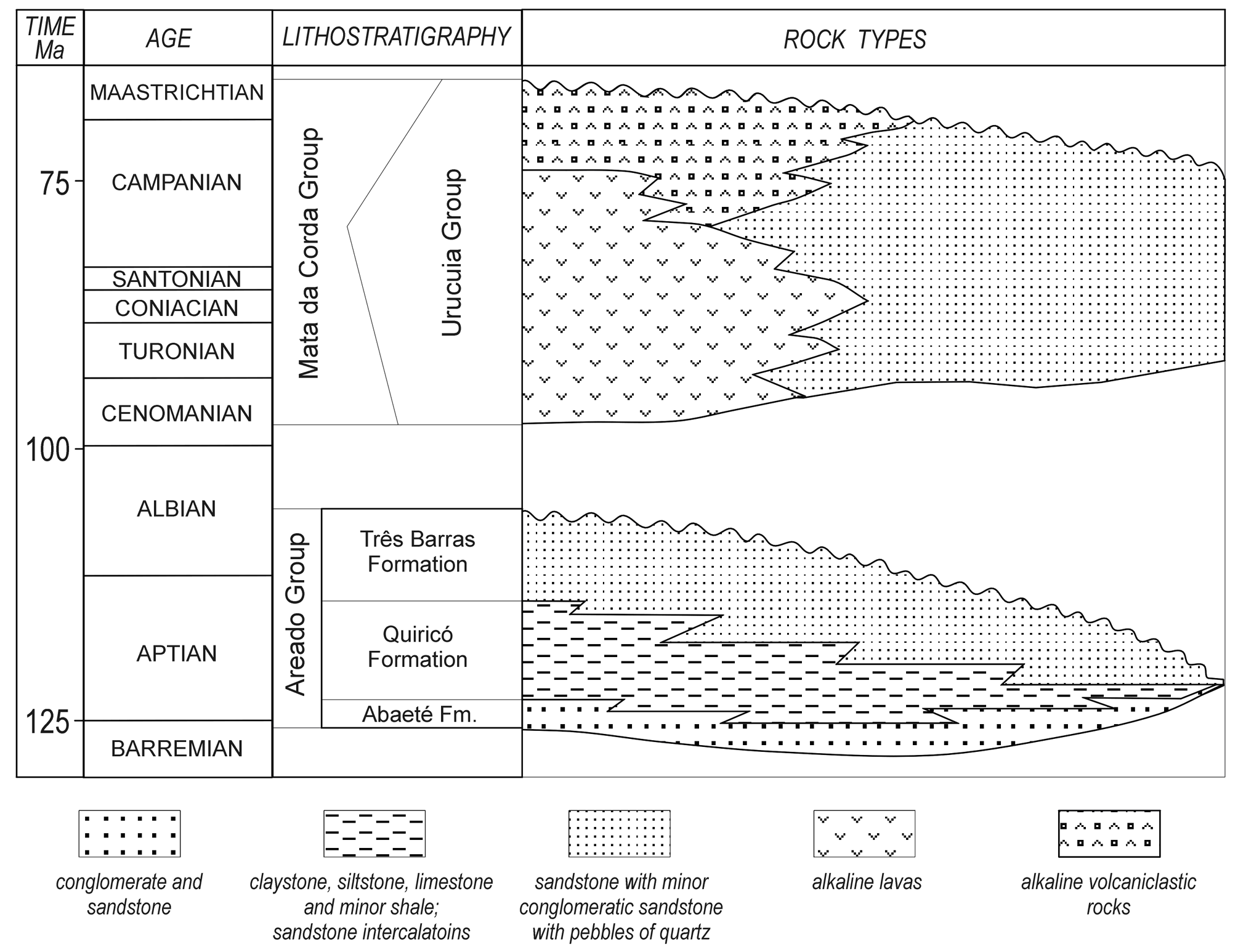

Supplement: Figure S2 — Stratigraphy of the Sanfranciscana basin. (TIF) [file pone.0016663.s002.tif]

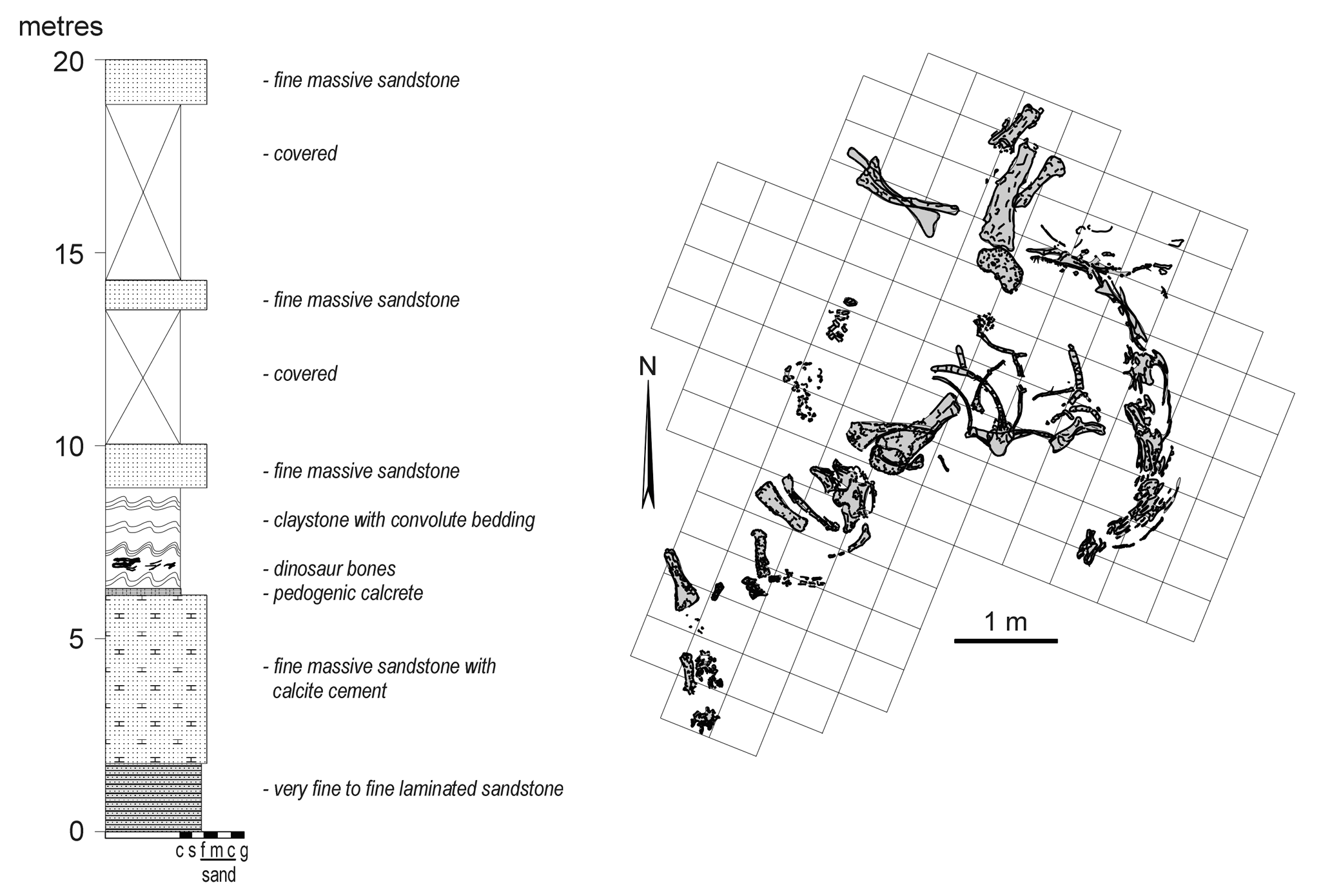

Supplement: Figure S3 — Columnar section and plan view of occurrence of fossil bones of Tapuiasaurus macedoi gen. n. sp. n. (TIF) [file pone.0016663.s003.tif]

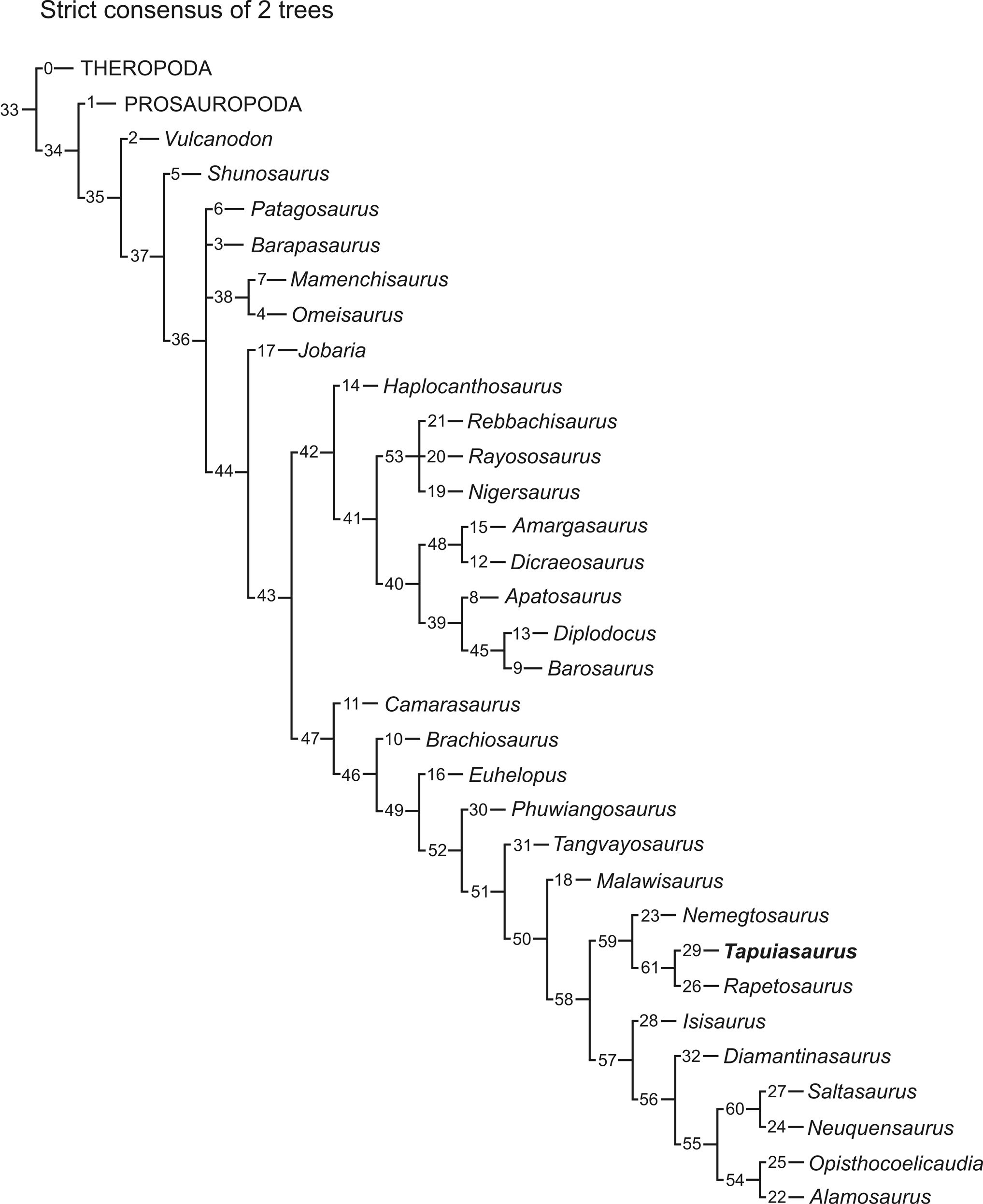

Supplement: Figure S4 — Strict consensus of the two most parsimonious trees found in the phylogenetic analysis. (TIF) [file pone.0016663.s004.tif]

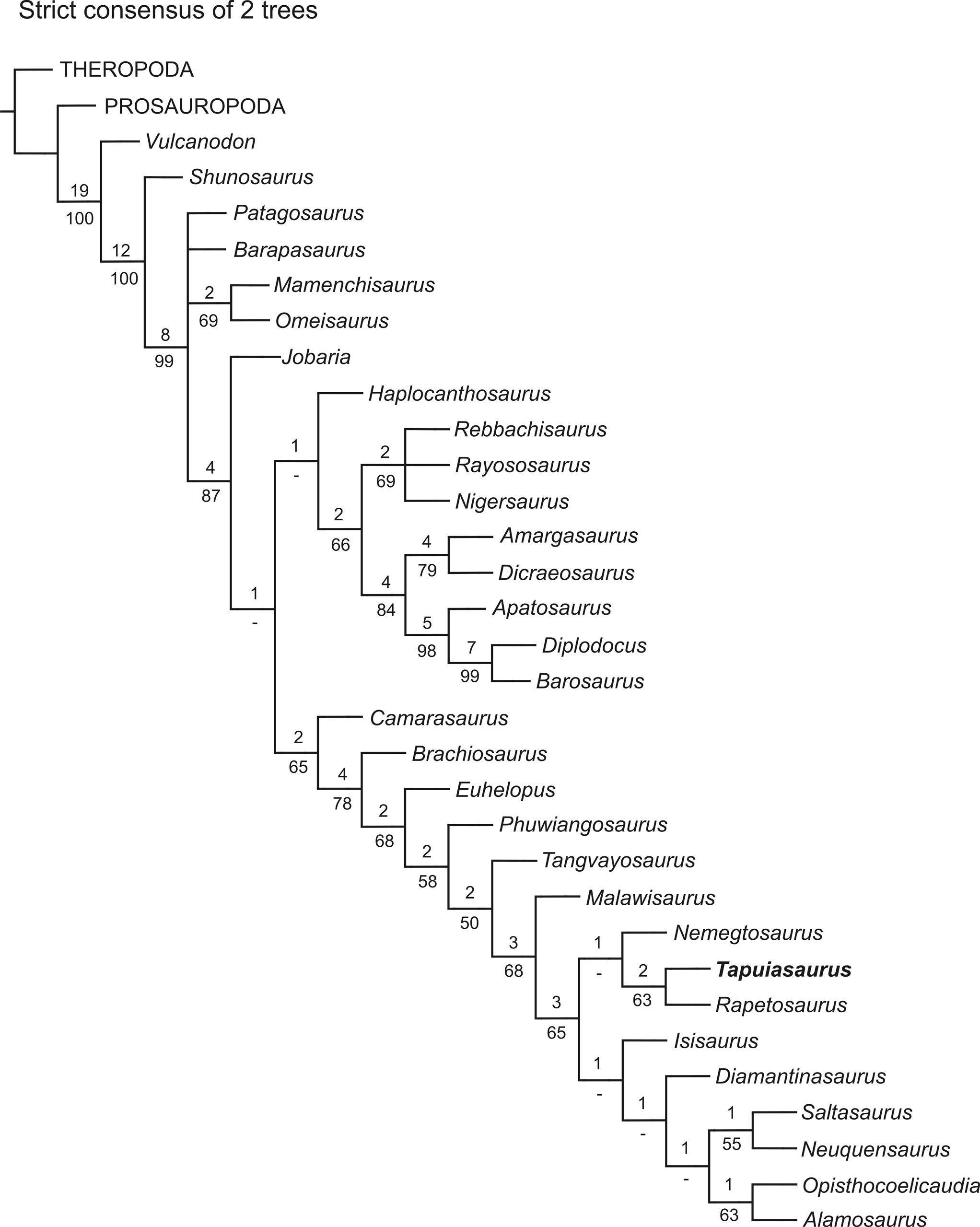

Supplement: Figure S5 — Bremer and bootstrap support values for the nodes of the consensus tree. (TIF) [file pone.0016663.s005.tif]

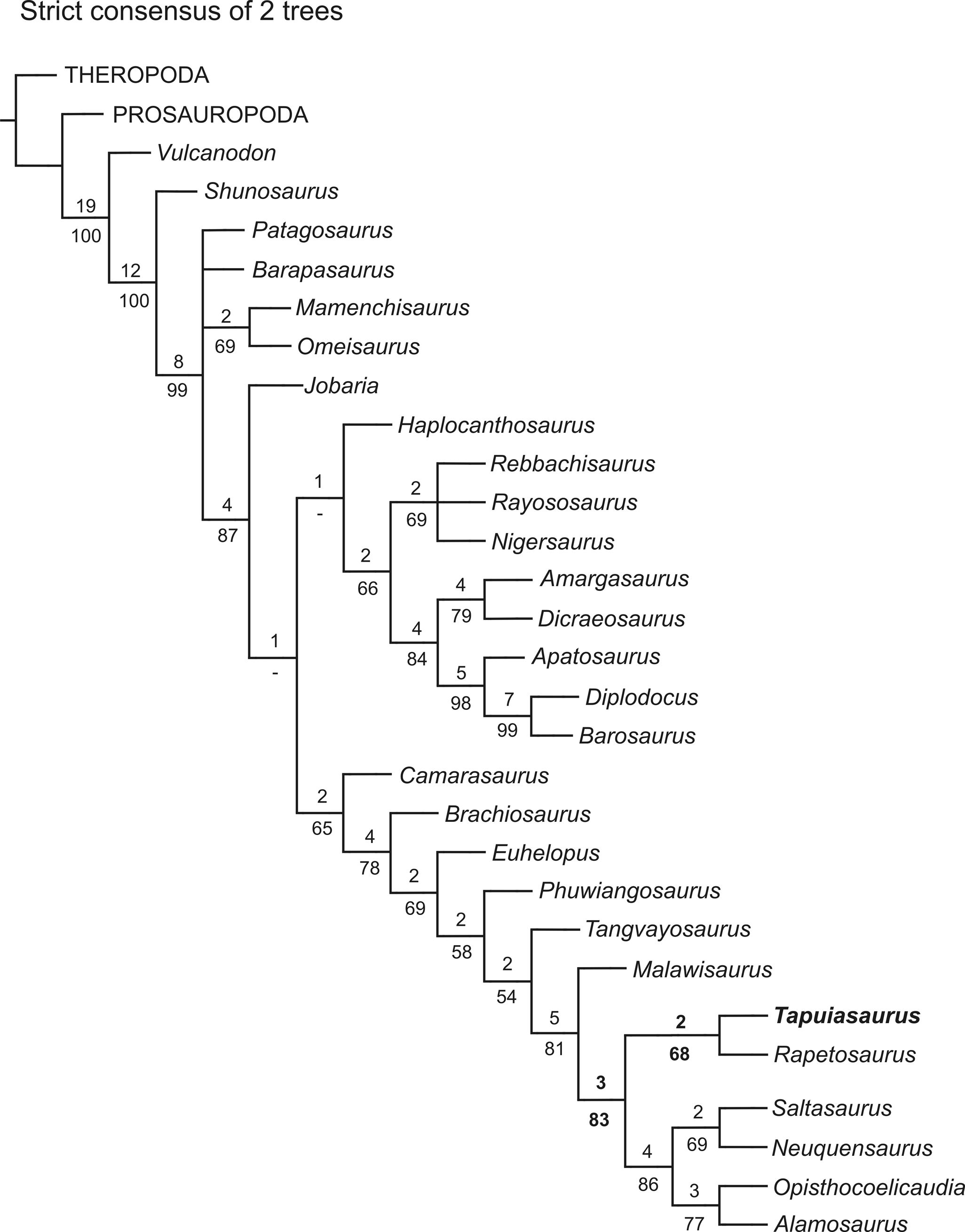

Supplement: Figure S6 — Bremer and bootstrap support values for the nodes on a reduced consensus tree. The tree shows support values that result from applying reduced consensus during the Bremer and Bootstrap analyses, ignoring the alternative positions of the most unstable advanced titanosaurians included in the analysis (i.e., Nemegtosaurus, Diamantinasaurus, and Isisaurus). (TIF) [file pone.0016663.s006.tif]
