# Supplementary material for: A Complete Skull of an Early Cretaceous Sauropod and the Evolution of Advanced Titanosaurians
Source: PLoS One. 2011 Feb 7;6(2):e16663. doi: 10.1371/journal.pone.0016663 (PMC3034730; doi:10.1371/journal.pone.0016663)
Supplement: Text S1 — Geological Setting. (DOC) [file pone.0016663.s007.doc]

**TEXT S1. GEOLOGICAL SETTING.**

The Sanfranciscana Basin is a large, N-S-oriented rift basin of eastern Brazil. With an approximate length of 1100 km and up to 270 km in width (Figure S1), the basin has a maximum preserved thickness of 360 m of sedimentary and volcanic rocks [1]. Although located about 500 km far from the ocean, the basin roughly parallels the present-day coastline, which reflects its tectonic control. Its installation was controlled by the activity of N-S-oriented listric normal faults [2,3] related to the regional E-W-oriented extension active during the Early Cretaceous and responsible for the Gondwana breakup and the opening of the South Atlantic Ocean.

The age of deposition in the Sanfranciscana Basin is well constrained by two magmatic events. The basin postdates the eruption of the Paraná continental flood basalts dated at the 138-128 Ma time-interval [4]. Alkalic ultrabasic hypabissal and volcanic rocks dated at 95-76 Ma time-interval [5-8] are coeval to the Late Cretaceous sedimentation, the volcanic rocks occurring intercalated with sandstones of the upper part of the basin fill [9].

The sedimentary and volcanic fill of the Sanfranciscana Basin is subdivided into the lower, Early Cretaceous Areado Group, and the upper, Late Cretaceous and laterally interfingered Mata da Corda (which includes alkaline lavas and volcaniclastic rocks) and Urucuia groups (Figure S2). In the studied region, the Early Cretaceous Areado Group comprises, from lower to top, the Abaeté, Quiricó and Três Barras Formations [10,11]. The Abaeté Formation is a thin, up to 8 m thick package of conglomerate and sandstone of aluvial fans to fluvial braided system [9]. The intermediate Quiricó Formation is a succession of lacustrine claystone, siltsone, limestone and minor shale with sandstone intercalations reaching up to 100 m in thickness [10]. The upper Três Barras Formation is a thick, up to 140 m succession of aeolian, fluvial-deltaic and fluvial meandering sandstone with minor conglomeratic sandstone [9], and with scarce centimetric peebles of quartz [12].

The skeleton of *Tapuiasaurus macedoi* gen. et sp. nov. was found in an outcrop of lacustrine claystone beds of the Quiricó Formation, at Embira-Branca hills near the town of Coração de Jesus, northern region of the state of Minas Gerais, Brazil (Figure S3). It was found associated with an articulated skeleton of an abelisauroid theropod.

Many occurrences of fossils were previously reported in the lacustrine deposits of the Quiricó Formation. Near Presidente Olegário, the fossil remains of the small teleostean fish *Dastilbe moraesi* [13], of Aptian age [14], were found in shale. The osteoglossiform fish *Laeliichthys ancentralis*, also of Aptian age [15], was reported in the same locality. Ostracods associated with pollen grais of the *Transistoripollis crisopolensis* palinozone indicated a post-Barremian, early Aptian age [16], as this pollen disappeared by the mid-Aptian [17]. The Quiricó Formation contains a high *Afropollis* count and include other angiosperm pollen grains that appeared at the Barremian–Aptian boundary [17]. Fifteen non-marine ostracod species were identified in samples of the Quiricó Formation from Carmo do Paranaíba and near Rio do Sono [18]. Three of them (*D. martinsi*, *Brasacypris* sp. 1, and *H. symmetrica*?) were previously recorded in Aptian deposits of other basins in Brazil whereas two others (*Wolburgiopsis plastica* and *Wolburgiopsis chinamuertensis*) are restricted to Barremian strata of Argentine [18]. Bones of the coelacanth fish *Mawsonia* found near the town of João Pinheiro were recently assigned to the Berriasian [19], but this age is incompatible with the post-Paraná flood volcanics (Hauterivian–Barremian) age of the Sanfransciscana Basin. Paleontological data therefore constrain the dinosaur-bearing beds of the Quiricó Formation to the Aptian.

**References (Text S1)**

1. Braun OPG (1970) A Formação Areado e a Formação Serra Negra. Rev. Esc. Minas Ouro Preto 28: 100-106.
2. Hasui Y, Haralyi NLE (1991) Aspectos lito-estruturais e geofísicos do Soerguimento do Alto Paranaíba. Geociências 10: 57-77.
3. Alkmin FF, Chemale Jr. F, Endo I (1996) A deformação das coberturas proterozóicas do Cráton do São Francisco e seu significado tectônico. Rev. Esc. Minas Ouro Preto 49: 22-38.
4. Turner S, Regelous M, Kelley S, Hawkesworth C, Mantovani M (1994) Magmatism and continental break-up in the South Atlantic: high precision ^40^Ar-^39^Ar geochronology. Earth Planet. Sci. Letters 124: 333-348.
5. Hasui Y, Cordani UG (1968) Idade Potássio-Argônio de rochas eruptivas Mesozóicas do Oeste Mineiro e sul de Goiás. In: Sociedade Brasileira de Geologia*,* Editor. Anais do 22^o^ Congresso Brasileiro de Geologia. Belo Horizonte: Sociedade Brasileira de Geologia. pp. 139-143.
6. Gibson SA, Thompson RN, Leonardos OH, Turner SE, Mitchell JG et al. (1994) The Serra do Bueno potassic diatreme: a possible hypabissal equivalent of the ultramafic alkaline volcanics in the Late Cretaceous Alto Paranaíba Igneous Province, Se Brazil. Mineral. Mag. 58: 357-373.
7. Sgarbi PBA, Heaman LM, Gaspar JC (2004) U–Pb perovskite ages for brazilian kamafugitic rocks: further support for a temporal link to a mantle plume hotspot track. J. South Amer. Earth Sci. 16: 715-724.
8. Riccomini C, Velázquez VF, Gomes CB (2005) Tectonic controls of the Mesozoic and Cenozoic alkaline magmatism in central-southeastern Brazilian Platform. In: Gomes CB, Comin-Chiaramonti P, Editors. Mesozoic to Cenozoic alkaline magmatism in the Brazilian Platform. São Paulo: EDUSP-FAPESP. pp. 31-55.
9. Sgarbi GNC, Sgarbi PBA, Campos JEG, Dardenne MA, Penha UC (2001) Bacia Sanfranciscana: o Registro Fanerozóico da Bacia do São Francisco. In: Pinto CP, Martins-Neto M, Editors. Bacia do São Francisco: Geologia e Recursos Naturais. Belo Horizonte: Sociedade Brasileira de Geologia. pp. 93-138.
10. Barbosa O (1965) Série Bambuí. Simpósio das Formações Eo-paleozóicas do Brasil. In: Sociedade Brasileira de Geologia, Editor. Anais do 19^o^ Congresso Brasileiro de Geologia. Rio de Janeiro: Sociedade Brasileira de Geologia. p. 11.
11. Campos JEG, Dardenne MA (1997) Estratigrafia e sedimentação da Bacia Sanfranciscana: uma revisão. Rev. Bras. Geociências 27: 269-282.
12. Grossi Sad JH, Cardoso RN, Costa MT (1971) Formações cretácicas em Minas Gerais: uma revisão. Rev. Bras. Geociências 1: 2-13.
13. Silva Santos R (1955) Descrição dos peixes fosseis. Bol. Min. Agr. Div. Geol. Mineral., Rio de Janeiro 155: 1-211.
14. Carvalho IS, Bertolino LC, Borghi LF, Duarte L, Carvalho MSS et al. (1994) The São Francisco Basin. In: Beurlen G, Campos DA, Viviers MC, Editors. Stratigraphic range of Cretaceous mega- and microfossils of Brazil. Rio de Janeiro: Universidade Federal do Rio de Janeiro. pp. 333-352.
15. Santos RS (1985) *Laeliichthys ancestralis*, novo gênero e espécie de Osteoglossiformes do Aptiano da Formação Areado, Estado de Minas Gerais, Brasil. Colet. Trab. Paleontol. Ser. Geol. 27: 161-167.
16. Arai M, Dino R, Milhomem PS, Sgarbi GNC (1995) Micropaleontologia de Formação Areado, Cretáceo da bacia Sanfranciscana: estudos de ostracodes e palinologia. In: Sociedade Brasileira de Paleontologia, Editor. Anais do 14^o^ Congresso Brasileiro de Paleontologia. Uberaba: Sociedade Brasileira de Paleontologia. pp. 2-3.
17. Maisey JG (2000) Continental break up and the distribution of fishes of Western Gondwana during the Early Cretaceous. Cret. Res. 21: 281-314.
18. Carmo DA, Tomassi HZ, Oliveira SBSG (2004) Taxonomia e distribuição estratigráfica dos ostracodes da Formação Quiricó, Grupo Areado (Cretáceo Inferior), bacia Sanfranciscana, Brasil. Rev. Bras. Paleontologia 7: 139-149.
19. Carvalho MSS, Maisey JG (2008) New occurrence of Mawsonia (Sarcopterygii: Actinistia) from the Early Cretaceous of the Sanfranciscana Basin, Minas Gerais, southeastern Brazil. In: Cavin L, Longbottom A, Richter M, Editors. Fishes and the Break-up of Pangaea. London: Geological Society Special Publications 295. pp. 109-144.
