# Supplementary material for: A Complete Skull of an Early Cretaceous Sauropod and the Evolution of Advanced Titanosaurians
Source: PLoS One. 2011 Feb 7;6(2):e16663. doi: 10.1371/journal.pone.0016663 (PMC3034730; doi:10.1371/journal.pone.0016663)
Supplement: Text S2 — Character list and data matrix used in phylogenetic analysis. Character definitions 1 to 234 are from [15] and have the same numeration as in the original publication. The additional characters are either new or taken from [2] and their respective sources are cited along with the character number of the original publication. Characters 8, 37, 64, 66, and 198 were set as ordered. The data matrix corresponds to the phylogenetic analysis published in [15] with the following modifications. Character scorings for Euhelopus were taken from the corrected list provided in [34]. Character scorings for Phuwiangosaurus and Tangvayosaurus were taken from [16] and those of Diamantinasaurus follow those given by [10]. (DOC) [file pone.0016663.s008.doc]

**TEXT S2. CHARACTER LIST AND DATA MATRIX USED IN PHYLOGENETIC ANALYSIS.**

**Character list**

Character definitions 1 to 234 are from [1] and have the same numeration as in the original publication. The additional characters are either new or taken from [2] and [3], and their respective sources are cited along with the character number of the original publication. Characters 8, 37, 64, 66, and 198 were set as ordered.

1. Posterolateral processes of premaxilla and lateral processes of maxilla, shape

0: without midline contact

1: with midline contact contact forming marked narial depression, subnarial foramen not visible laterally

2. Premaxillary anterior margin, shape

0: without step

1: with marked step, anterior portion of skull sharply demarcated

3. Maxillary border of external naris, length

0: short, making up much less than one-fourth narial perimeter

1: long, making up more than one-third narial perimeter

4. Preantorbital fenestra

0: absent

1: present

5. Subnarial foramen and anterior maxillary foramen, position

0: well distanced from one another

1: separated by narrow bony isthmus

6. Antorbital fenestra, maximum diameter

0: much shorter than orbital maximum diameter

1: subequal to orbital maximum diameter

7. Antorbital fossa

0: present

1: absent

8. External nares, position

0: terminal

1: retracted to level of orbit

2: retracted to a position between the orbits

9. External nares, maximum diameter

0: shorter than orbital diameter

1: longer than orbital diameter

10. Orbital ventral margin, anteroposterior length

0: broad, with subcircular orbital margin

1: reduced, with acute margin

11. Lacrimal anterior process

0: present

1: absent

12. Jugal-ectopterygoid contact

0: present

1: absent

13. Jugal, contribution to antorbital fenestra

0: very reduced or absent

1: large, borderin approximately one-third its parimeter

14. Prefrontal, posterior process size

0: small, not projecting far posterior of frontal-nasal suture

1: elongate, approaching parietal

15. Prefrontal posterior process shape

0: flat

1: hooked

16. Postorbital, ventral process shape

0: transversely narrow

1: broader transversely than anteroposteriorly

17. Postorbital, posterior process

0: present

1: absent

18. Frontal contribution to supratemporal fossa

0: present

1: absent

19. Frontals, midline contact (symphysis)

0: sutured in adult individuals

1: fused in adult individuals

20. Frontal, anteroposterior length

0: approximately twice minimum transvers breadth

1: less than minimum transverse breadth

21. Parietal occipital process, dorsoventral height

0: short, less than the diameter of the foramen magnum

1: deep, nearly twice the diameter of the foramen magnum

22. Parietal contribution to post-temporal fenestra

0: present

1: absent

23. Postparietal foramen

0: absent

1: present

24. Parietal, distance separating supratemporal fenestrae

0: less than the long axis of supratemporal fenestra

1: twice the long axis of supratemporal fenestra

25. Supratemporal fenestra

0: present

1: absent

26. Supratemporal fenestra, long axis orientation

0: anteroposterior

1: transverse

27. Supratemporal fenestra, maximum diameter

0: much longer than that of the foramen magnum

1: subequal to that of the foramen magnum

28. Supratemporal region, anteoposterior length

0: temporal bar longer anteroposteriorly than transversely

1: temporal bar shorter anteroposteriorly than transversely

29. Supratemporal fossa, lateral exposure

0: not visible laterally, obscured by temporal bar

1: visible laterally, temporal bar shifted ventrally

30. Laterotemporal fenestra, anterior extension

0: posterior to orbit

1: ventral to orbit

31. Squamosal-quadratojugal contact

0: present

1: absent

32. Quadratojugal, anterior process length

0: short, anterior process shorter than dorsal process

1: long, anterior process more than twice as long as dorsal process

33. Quadrate fossa

0: absent

1: present

34. Quadrate fossa, depth

0: shallow

1: deeply invaginated

35. Quadrate fossa, orientation

0: posterior

1: posterolateral

36. Palatobasal contact, shape

0: pterygoid with small facet

1: dormedially oriented hook

2: rocker-like surface for basipterygoid articulation

37. Pterygoid, transverse flange (ie, ectopterygoid process) position

0: posterior of orbit

1: between orbit and antorbital fenestra

2: anterior to antorbital fenestra

38. Pterygoid, quadrate flange size

0: large, palatobasal and quadrate articulations well separated

1: small, palatobasal and quadrate articulations approach

39. Pterygoid, palatine ramus shape

0: straight, at level of dorsal margin of quadrate ramus

1: stepped, raised above level of quadrate ramus

40. Palatine lateral ramus shape

0: plate-shaped (long maxillary contact)

1: rod-shaped (narrow maxillary contact)

41. Epipterygoid

0: present

1: absent

42. Vomer, anterior articulation

0: maxilla

1: premaxilla

43. Supraoccipital height

0: twice the foramen magnum height

1: subequal or less than the foramen magnum height

44. Paroccipital process, ventral nonarticular process

0: absent

1: present

45. Crista prootica, size

0: rudimentary

1: expanded laterally into "dorsolateral process"

46. Basipterygoid process length

0: short, approximately twice the basal diameter

1: elongated, at least four times the basal diameter

47. Basipterygoid process, angle of divergence

0: approximately 45 degrees

1: less than 30 degrees

48. Basal tubera, anteroposterior depth

0: approximately half dorsoventral height

1: sheet-like, 20 % dorsoventral height

49. Basal tubera, breadth

0: much broader than occipital condyle

1: narrower than occipital condyle

50. Basioccipital depression between foramen magnum and basal tubera

0: absent

1: present

51. Basisphenoid-basipterygoid recess

0: present

1: absent

52. Basisphenoid-quadrate contact

0: absent

1: present

53. Basipterygoid process, orientation

0: perpendicular to skull roof

1: angled approximately 45 degrees to skull roof

54. Occipital region of skull, shape

0: anteroposteriorly deep, paroccipital processes oriented posterolaterally

1: flat, paroccipital processes oriented transversely

55. Dentary, depth of anterior end of ramus

0: slightly less than that of dentary at midlength

1: 150% minimum depth

56. Dentary, anteroventral margin shape

0: gently rounded

1: sharply projecting triangular process or 'chin'

57. Dentary symphysis, orientation

0: angled 15 degrees or more anteriorly to axis of jaw ramus

1: perpendicular to axis of jaw ramus

58. External mandibular fenestra

0: present

1: absent

59. Surangular depth

0: less than twice maximum depth of the angular

1: more than two and one-half times maximum depth of the angular

60. Surangular ridge separating adductor and articular fossae

0: absent

1: present

61. Adductor fossa, medial wall depth

0: shallow

1: deep, prearticular expanded dorsoventrally

62. Splenial posterior process, position

0: overlapping angular

1: separating anterior portions of prearticular and angular

63. Splenial posterodorsal process

0: present, approaching margin of adductor chamber

1: absent

64. Coronoid, size

0: extending to dorsal margin of jaw

1: reduced, not extending dorsal to splenial

2: absent

65. Tooth rows, shape of anterior portions

0: narrowly arched, anterior portion of tooth rows V-shaped

1: broadly arched, anterior portion of tooth rows U-shaped

2: rectangular, tooth-bearing portion of jaw perpendicular to jaw rami

66. Tooth rows, length

0: extending to orbit

1: restricted anterior to orbit

2: restricted anterior to subnarial foramen

67. Crown-to-crown occlusion

0: absent

1: present

68. Occlusal pattern

0: interlocking, V-shaped facets

1: high-angled planar facets

2: low-angled planar facets

69. Tooth crowns, orientation

0: aligned along jaw axis, crowns do not overlap

1: aligned slightly anterolingually, tooth crowns overlap

70. Tooth crowns, cross-sectional shape at mid-crown

0: elliptical

1: D-shaped

2: cylindrical

71. Enamel surface texture

0: smooth

1: wrinkled

72. Marginal tooth denticles

0: present

1: absent on posterior edge

2: absent on both anterior and posterior edges

73. Dentary teeth, number

0: greater than 20

1: 17 or fewer

74. Replacement teeth per alveolus, number

0: two or fewer

1: more than four

75. Teeth, orientation

0: perpendicular to jaw margin

1: oriented anteriorly relative to jaw margin

76. Teeth, longitudinal grooves on lingual aspect

0: absent

1: present

77. Presacral bone texture

0: solid

1: spongy, with large, open internal cells, "camellate"

78. Presacral centra, pneumatopores

0: absent

1: present

79. Atlantal intercentrum, occipital facet shape

0: rectangular in lateral view, length of dorsal aspect subequal to that of ventral aspect

1: expanded anteroventrally in lateral view, anteroposterior length of dorsal aspect shorter than that of ventral aspect

80. cervical vertebrae number

0: 9 or fewer

1: 10

2: 12

3: 13

4: 15 of greater

81. Cervical neural arch lamination

0: well developed, with well defined laminae and coels

1: rudimentary; diapophyseal laminae only feebly developed if present

82. Cervical centra, articular face morphology

0: amphicoelous

1: opisthocoelous

83. Cervical pneumatophores (pleurocoels), shape

0: simple, undivided

1: complex, divided by bony septa

84. Anterior cervical centra, height-width ratio

0: less than 1

1: approximately 1.25

85. Anterior cervical neural spines, shape

0: single

1: bifid

86. Mid-cervical centra, anteroposterior length-height of posterior face

0: 2.5 to 3.0

1: superior to 4

87. Mid-cervical neural arches, height

0: less than that of posterior centrum face

1: greater than that of posterior centrum face

88. Middle and posterior cervical neural arches, centroprezygapophyseal lamina (cprl), shape

0: single

1: divided

89. Posterior cervical and anterior dorsal neural spines, shape

0: single

1: bifid

90. Posterior cervical and anterior dorsal bifid neural spines, median tubercle

0: absent

1: present

91. Dorsal vertebrae, number

0: 15

1: 14

2: 13

3: 12

4: 11

5: 10 or fewer

92. Dorsal neural spines, breadth

0: narrower transversely than anteroposteriorly

1: much broader transversely than anteroposteriorly

93. Dorsal neural spines, length

0: approximately twice centrum length

1: approximately four times centrum length

94. Anterior dorsal centra, articular face shape

0: amphicoelous

1: opisthocoelous

95. Middle and posterior dorsal neural arches, centropostzygapophyseal lamina (cpol), shape

0: single

1: divided

96. Middle and posterior dorsal neural arches, anterior centroparapophyseal lamina (acpl)

0: absent

1: present

97. Middle and posterior dorsal neural arches, prezygoparapophyseal lamina (prpl)

0: absent

1: present

98. Middle and posterior dorsal neural arches, posterior centroparapophyseal lamina (pcpl)

0: absent

1: present

99. Middle and posterior dorsal neural arches, spinodiapophyseal lamina (spdl)

0: absent

1: present

100. Middle and posterior dorsal neural arches spinopostzygapophyseal lamina (spol) shape

0: single

1: divided

101. Middle and posterior dorsal neural arches, spinodiapophyseal lamina (spdl) and spinopostzygapophyseal lamina (spol) contact

0: absent

1: present

102. Middle and posterior dorsal neural spines, shape

0: tapering or not flaring distally

1: flared distally, with pendant, triangular lateral processes

103. Middle and posterior dorsal neural arches, infradiapophyseal pneumatopore between acdl and pcdl

0: absent

1: present

104. Middle and posterior dorsal neural spines, orientation

0: vertical

1: posterior, neural spine summit approaches level of diapophyses

105. Posterior dorsal centra, articular face shape

0: amphicoelous

1: opisthocoelous

106. Posterior dorsal neural arches, hyposphene-hypantrum articulations

0: present

1: absent

107. Posterior dorsal neural spines, shape

0: rectangular through most of length

1: petal shaped, expanding transversely through 75% of its length and then tapering

108. Sacral vertebrae, number

0: 3 or fewer

1: 4

2: 5

3: 6

109. Sacrum, sacricostal yoke

0: absent

1: present

110. Sacral vertebrae contributing to acetabulum

0: numbers 1-3

1: numbers 2-4

111. Sacral neural spines, length

0: approximately twice length of centrum

1: four times length of centrum

112. Sacral ribs, dorsoventral length

0: low, not projecting beyond dorsal margin of ilium

1: high, extending beyond dorsal margin of ilium

113. Caudal bone texture

0: solid

1: spongy, with large internal cells

114. Caudal vertebrae, number

0: more than 45

1: 35 or fewer

115. Caudal transverse processes

0: persist through caudal 20 or more posteriorly

1: disappear by caudal 15

2: disappear by caudal 10

116. First caudal centrum, articular face shape

0: flat

1: procoelous

2: opistocoelous

3: biconvex

117. First caudal neural arch, coel on lateral aspect of neural spine

0: absent

1: present

118. Anterior caudal centra (excluding the first), articular face shape

0: amphyplatian or platycoelous

1: procoelous

2: opisthocoelous

119. Anterior caudal centra, pneumatophores (pleurocoels)

0: absent

1: present

120. Anterior caudal centra, length

0: approximately the same

1: doubling

2: over the first 20 vertebrae

121. Anterior caudal neural arches, spinozygaphophyseal laminae (sprl)

0: absent

1: present and extending onto lateral aspect of neural spine

122. Anterior caudal neural arches, spinoprezygapophyseal laminae (sprl)-spinozygapophyseal lamina (spol) contact

0: absent

1: present, forming a prominent lamina on lateral aspect of neural spine

123. Anterior caudal neural arches, prespinal lamina (prsl)

0: absent

1: present

124. Anterior caudal neural arches, postspinal lamina (posl)

0: absent

1: present

125. Anterior caudal neural arches, postspinal fossa

0: absent

1: present

126. Anterior caudal neural spines, transverse breadth

0: approximately 50% of anteroposterior length

1: greater than anteroposterior length

127. Anterior caudal transverse processes, proximal depth

0: shallow, on centrum only

1: deep, extending from centrum to neural arch

128. Anterior caudal transverse processes, shape

0: triangular, tapering distally

1: wing-like, not tapering distally

129. Anterior caudal transverse processes, diapophyseal laminae (acdl, pcdl, prdl, podl)

0: absent

1: present

130. Anterior caudal transverse processes, anterior centrodiapophyseal lamina (acdl), shape

0: single

1: divided

131. Anterior and middle caudla centra, shape

0: cylindrical

1: quadrangular, flat ventrally and laterally

132. Anterior and middle caudal centra, ventral longitudinal hollow

0: absent

1: present

133. Middle caudal neural spines, orientation

0: angled posterodorsally

1: vertical

134. Middle and posterior caudal centra, anterior articular face shape

0: flat

1: procoelous (cone shaped)

2: opisthocoelous

135. Posterior caudal centra, shape

0: cylindrical

1: dorsoventrally flattened, breadth at least twice height

136. Distalmost caudal cantra, articular face shpae

0: platycoelous

1: biconvex

137. Distalmost biconvex caudal centra, length-to-height ratio

0: less than four

1: greater than five

138. Distalmost biconvex caudal centra, number

0: 10 or fewer

1: more than 30

139. Cervical rib, tuberculum-capitulum angle

0: greater than 90 degrees

1: less than 90 degrees, rib ventrolateral to centrum

140. Cervical ribs, length

0: much longer than centrum, overlapping as many as three subsequent vertebrae

1: shorter than centrum, little or no overlap

141. Dorsal ribs, proximal pneumatocoels

0: absent

1: present

142. Anterior dorsal ribs, cross-sectional shape

0: subcircular

1: plank-like, anteroposterior breadth more than three times mediolateral breadth

143. Forked chevrons with anterior and posterior projections

0: absent

1: present

144. Forked chevrons, distribution

0: distal tail only

1: throughout middle and posterior caudal vertebrae

145. Chevrons, crus bridging dorsal margin of haemal canal

0: present

1: absent

146. Chevron haemal canal, depth

0: short, approximately 25% chevron length

1: long, approximately 50% chevron length

147. Chevrons

0: persisting throughout at least 80% of tail

1: disappearing by caudal 30

148. Posterior chevrons, distal contact

0: fused

1: unfused (open)

149. Posture

0: bipedal

1: columnar, obligately quadruped posture

150. Scapular acromion process, size

0: narrow

1: broad, width more than 150% minimum width of blade

151. Scapular blade, orientation

0: perpendicular to coracoid articulation

1: forming a 45 degree angle with coracoid articulation

152. Scapular blade, shape

0: acromial edge not expanded

1: rounded expansion on acromial side

2: racquet-shaped

153. Scapular glenoid, orientation

0: relatively flat or laterally facing

1: strongly bevelled medially

154. Scapular blade, cross-sectional shape at base

0: flat or rectangular

1: D-shaped

155. Coracoid, proximodistal length

0: less than length of scapular articulation

1: approximately twice the length of scapular articulation

156. Coracoid, anteroventral margin shape

0: rounded

1: rectangular

157. Coracoid, infraglenoid lip

0: absent

1: present

158. Sternal plate, shape

0: oval

1: crescentric

159. Humeral proximolateral corner, shape

0: rounded

1: square

160. Humeral deltopectoral attachment, development

0: prominent

1: reduced to a low crest or ridge

161. Humeral deltopectoral crest, shape

0: relatively narrow throughout length

1: markedly expanded distally

162. Humeral midshaft cross-section, shape

0: circular

1: elliptical, with long axis orientated transversely

163. Humeral distal condyles, articular surface shape

0: restricted to distal portion of humerus

1: exposed on anterior portion of humeral shaft

164. Humeral distal condyle, shape

0: divided

1: flat

165. Ulnar proximal condyle, shape

0: subtriangular

1: triradiate, with deep radial fossa

166. Ulnar proximal condylar processes, relative lengths

0: subequal

1: unequal, anterior arm longer

167. Ulnar olecranon process, development

0: prominent, projecting above proximal articulation

1: rudimentary, level with proximal articulation

168. Ulna, length-to-proximal breadth ratio

0: gracile

1: stout

169. Radial distal condyle, shape

0: round

1: subrectangular, flattened posteriorly and articulating in front of ulna

170. Radius distal breadth

0: slightly larger than midshaft breadth

1: approximately twice than midshaft breadth

171. Radius, distal condyle orientation

0: perpendicular relative to long axis of shaft

1: bevelled approximately 20 degrees proximolaterally relative to long axis of shaft

172. Humerus-to-femur ratio

0: less than 0.60

1: 0.60 or more

173. Carpal bones, number

0: 3 or more

1: 2 or fewer

174. Carpal bones, shape

0: round

1: block-shaped, with flattened proximal and distal surfaces

175. Metacarpus, shape

0: spreading

1: bound, with subparallel shafts and articular surfaces that extend half their length

176. Metacarpals, shape of proximal surface in articulation

0: gently curving, forming a 90 degree arc

1: shaped, subtending a 270 degree arc

177. Longest metacarpal-to-radius ratio

0: close to 0.3

1: 0.45 or more

178. Metacarpal I, length

0: shorter than metacarpal IV

1: longer than metacarpal IV

179. Metacarpal I, distal condyle shape

0: divided

1: undivided

180. Metacarpal I distal condyle, transverse axis orientation

0: bevelled approximately 20 degrees proximo-distally with respect to axis of shaft

1: perpendicular with respect to axis of shaft

181. Manual digits II and III, phalangeal number

0: 2-3-3-2 or more

1: reduced, 2-2-2-2-2 or less

2: absent or unossified

182. Manual phalanx I-1, shape

0: rectangular

1: wedge-shaped

183. Manual nonungual phalanges, shape

0: longer proximodistally than broad transversely

1: broader transversely than long proximodistally

184. Pelvis, anterior breadth

0: narrow, ilia longer anteriposteriorly than distance separating preacetabular processes

1: broad, distance between preacetabular processes exceeds anteroposterior length of ilia

185. Ilium, ischial peduncle size

0: large, prominent

1: low, rounded

186. Iliac blade dorsal margin, shape

0: flat

1: semicircular

187. Iliac preacetabular process, orientation

0: anterolateral to body axis

1: perpendicular to body axis

188. Iliac preacetabular process, shape

0: pointed, arching ventrally

1: semicircular, with posteroventral excursion of cartilage cap

189. Pubis, ambiens process development

0: small, confluent with anterior margin of pubis

1: prominent, projecting anteriorly from anterior margin of pubis

190. Pubic apron, shape

0: flat (straight sympysis)

1: canted anteromedially (gentle S-shaped symphysis)

191. Puboischial contact, length

0: approximately one-third total length of pubis

1: one-half total length of pubis

192. Ischial blade, length

0: much shorter than pubic blade

1: equal to or longer than pubic blade

193. Ischial blade, shape

0: emarginate distal to pubic peduncle

1: no emargination distal to pubic peduncle

194. Ischial distal shaft, shape

0: triangular, depth of ischial shaft increases medially

1: bladelike, medial and lateral depths subequal

195. Ischial distal shafts, cross-sectional shape

0: V-shaped, forming an angle of nearly 50 degree with each other

1: flat, nearly coplanar

196. Femoral fourth trocanther, development

0: prominent

1: reduced to crest or ridge

197. Femoral lesser trochanter,

0: present

1: absent

198. Femoral midshaft, transverse diameter

0: subequal to anteroposterior diameter

1: at least 125-150% to anteroposterior diameter

2: at least 185% to anteroposterior diameter

199. Femoral shaft, lateral margin shape

0: straight

1: proximal one-third deflected medially

200. Femoral distal condyles, relative transverse breadth

0: subequal

1: tibial much broader than fibular

201. Femoral distal condyles, orientation

0: perpendicular or slightly bevelled dorsolaterally relative to femoral shaft

1: bevelled dorsomedially approximately 10 degrees relative to femoral shaft

202. Femoral distal condyles, articular surface shape

0: restricted to distal portion of femur

1: expanded onto anterior portion of femoral shaft

203. Tibial proximal condyle, shape

0: narrow, long axis anteroposterior

1: expanded transversely, condyle subcircular

204. Tibial cnemial crest, orientation

0: projecting anteriorly

1: projecting laterally

205. Tibia, distal breadth

0: approximately 125%

1: more than twice midshaft breadth

206. Tibial distal posteroventral process, size

0: broad transversely, covering posterior fossa of astragalus

1: shortened transversely, posterior fossa of astragalus visible posteriorly

207. Fibula, proximal tibial scar, development

0: not well-marked

1: well-marked and deepening anteriorly

208. Fibula, lateral trochanter

0: absent

1: present

209. Fibular distal condyle, size

0: subequal to shaft

1: expanded transversely, more than twice mid-shaft breadth

210. Astragalus, shape

0: rectangular

1: wedge-shaped, with reduced anteromedial corner

211. Astragalus, foramina at base of ascending process

0: present

1: absent

212. Astragalus, ascending process length

0: limited to anterior two-thirds of astragalus

1: extending to posterior margin of astragalus

213. Astragalus, posterior fossa shape

0: undivided

1: divided by vertical crest

214. Astragalus, transverse length

0: 50% more than proximodistal height

1: subequal to proximodistal height

215. Calcaneum

0: present

1: absent or unossified

216. Distal tarsals 3 and 4

0: present

1: absent or unossified

217. Metatarsus, posture

0: bound

1: spreading

218. Metatarsal I proximal condyle, transverse axis orientation

0: perpendicular to axis of shaft

1: angled ventrallomedially approximately 15 degree to axis of shaft

219. Metatarsal I distal condyle, transverse axis orientation

0: perpendicular to axis of shaft

1: angled dorsomedially to axis of shaft

220. Metatarsal I distal condyle, posterolateral projection

0: absent

1: present

221. Metatarsal I, minimum shaft width

0: less than that of metatarsall II-IV

1: greater than that of metatarsal II-IV

222. Metatarsal I and V proximal condyle, size

0: smaller than those of metatarsals II and IV

1: ubequal to those of metatarsals II and IV

223. Metatarsal III length

0: more than 30% that of tibia

1: less than 25% that of tibia

224. Metatarsals III and IV, minimum transverse shaft diameters

0: subequal to that of metatarsals I and II

1: less than 65% that of metatarsals I and II

225. Metatarsal V, length

0: shorter than length of metatarsal IV

1: at least 70% length of metatarsal IV

226. Pedal nonungual phalanges, shape

0: longer proximodistally than broad transversely

1: broader than long proximodistally

227. Pedal digits II-IV, penultimate phalanges, development

0: subequal in size to more proximal phalanges

1: rudimentary or absent

228. Pedal unguals, orientation

0: aligned with digit axis

1: deflected lateral to digit axis

229. Pedal digit I ungual, length relative to pedal digit II ungual

0: subequal

1: 25% larger than that of digit II

230. Pedal digit I ungual, length

0: shorter than metatarsal I

1: longer than metatarsal I

231. Pedal ungual I, shape

0: broader transversely than dorsoventrally

1: sickle-shaped, much deeper dorsoventrally than broad transversely

232. Pedal ungual II-III, shape

0: broader transversely than dorsoventrally

1: sickle-shaped, much deeper dorsoventrally than broad transversely

233. Pedal digit IV ungual, development

0: subequal in size to unguals of pedal digits II and III

1: rudimentary or absent

234. Osteoderms

0: absent

1: present

235. maxilla, jugal process [2: character 9]

0: robust, broadly contacting the jugal

1: tapering posteriorly

236. Ventral edge of anterior surface of the quadratojugal [3: character 26]

0: straigth, not expanded ventrally

1: concave due to a ventral expansion of the anterior region

237. Lacrimal, anterior process

0: short, less than 50% of the length of the ventral process

1: long, at least 75% of the length of the ventral process

238. Prefrontal, anterior process [2: character 30]

0: absent

1: present

239. Prefrontal, width at the level of the frontal contact

0: large, equal or longer that the anteroposterior length of the prefrontal

1: narrow, less than half the anteroposterior length of the prefrontal

240. Pterygoid, sutural contact with ectopterygoid [1]

0: on the lateral surface of the ectopterygoid

1: on the medial surface of the ectopterygoid

241. Pterygoid, sutural contact with ectopterygoid

0: broad, along the medial or lateral surface

1: narrow, restricted to the anterior tip of the ectopterygoid

242. Basisphenoid, sagital ridge between basipterygoid processes

0: absent

1: present

243. Squamosal, participation in supratemporal fenestra [2: character 37]

0: present

1: absent

244. Maxilla, foramen anterior to the preantorbital fenestra

0: absent

1: present

245. Postorbital, posterior margin articulating with the squamosal [1: appendix 4]

0: with tapering posterior process

1: with a deep posterior process

246. Preantorbital fenestra, deep and large anteroposteriorly oriented fossa

0: opening directly on the lateral side of the maxilla or recessed in a small and shallow fossa

1: recessed into a deep and large anteroposteriorly oriented fossa

**Data Matrix**

The data matrix corresponds to the phylogenetic analysis published in [1] with the following modifications. Character scorings for *Euhelopus* were taken from the corrected list provided in [4]. Character scorings for *Phuwiangosaurus* and *Tangvayosaurus* were taken from [5] and those of *Diamantinasaurus* follow those given by [6].

PROSAUROPODA 000000000000000000000000000000000??00000000000000000000000000000000?00000000000110?000000?1000000000000000000?00000000000000000000000000??00000?000000000000000001000?00000000000100000000000000000000000000000000000000000000000000000000001100000?0?

THEROPODA 000000000000000000000000000000000??00000000000000000000000000000000?00000000000010?000000?0000000000000000000?00000000000000000000000000??00000?000001000000000001100?0000000000000000000000000000000000000000000000000000??00000000000000001100000?0?

Vulcanodon ????????????????0????????????????????????????????????????????????????????????0?????????????????????????????1????0??0?00???????100001????????????00??10?????????1010?11101001????0???????1???00010101010???000?00001000010?100100100?011000????????????

Barapasaurus ?????????????????????????????????????????????????????????????????????1?0????00??01???0100??10101101110100001100?0??0?00???????1000000000?????01?00?0110000000?0101011110100????????????01100010101011101000101111?1?10???010???????1??11?0????????????

Omeisaurus 11100011011?000?01011000010111?11?0?1??11100?0?0?0?00110000?1???111011111?0001040111010?0?3101?11011100000021101?010000000000010000?000???100?11000011000?00000101011010100101000000111?110001010101?1000?01011??0101001111111111111111110????????0?0?

Shunosaurus 01100011011000000001??000001110110001001100000000?000110000?101?111011120?00000311?100100?210000?00000000?010?000010000000000010000?000???0?00111010100000000001010110101001010000011010110001010?01110100?101?10010?00111101110111?111110000????00?0?

Patagosaurus 1?100?11????????0????????????????????????1?????????????0????????1?10111???0001??011?00100??10101101??0100002??0??????00???????1???0?000???1?00??00??110?0?000?0101111110100?????????????1100010101011101000001??????????101011???????????0????????????

Mamenchisaurus ????????????????????????????????110???????????????????10000?10111?1?1111000011?401?101011031010?1?1?10?00002?????011010000000010000?000???10001100?01?????????01010111101001????????????1100????01011101001101?1101110?11?????1?11?1??1??0??????????0?

Apatosaurus 00111112011?111?010111?101011111100020?1?0000100000011??????????221202121???011401101011115101111111100000021?110011000011110111110000011111001100101100110000010101101010011111000011111100110100011101001101110111101111111111?1111?111000000??00?0?

Barosaurus ????????????????????????????????????????????????????????????????????????????01??011?111?11?101????111000000?????0?11011111110111111110011????01?00?01????????0??????????????????????????????11???????????????????????????????????????????0????????????

Brachiosaurus 11110011111100010101100001011111110010111100000001100110011111121110011210001103011001100?31010111111100100211010?100000001100100000000???1011??10??110101000001010111101001111111111011110101110111111100110111111110011110111?11?1??11?00000000?0000

Camarasaurus 1111001111110001010110010101110011011011110000000010011001111111111011121000010201101010103101011011110010021101001000000011011000000000??100?10101011010100000101011110100111111100111111000111011111010011011101111001111011111111111110000000000000

Dicraeosaurus 00?11?????0??00?01111111011111?????1?0??10001110101011110???????221202121?00011201101010113111110011100000121?1?0?11000010110111000000011?11001?00??110001000?0101011110???1???????????1110011010001110100110111011110??111111?11????11??0????????0???

Diplodocus 001111120111111101011101010111111000201110000100001011110101101?221202121110011401101111115101111111100000021111001101111111011111111001111100110010110001000001010111101001???????????11100110100011101011101?101111001111111111111?11110000001100000

Haplocanthosaurus ????????????????0???????????????????????????????0???????????????????????????01?3011000100?21010110111100000211010?10000000110010000000????1100??10??100001000??????????????????????????1110001010111110100???????????????????????????????0????????????

Amargasaurus ??????????1??00101111?1101111?????????????00111011?011??????????????????????001301??1010105111????1??0000?12??1????0?????????????????0???????0??????1??0?1????0101011010?001????????????1100???????1110?00???????????????????????????????0????????0???

Euhelopus 01110?11??1????10???????0???????11???011??????????????1001??????111011121?1011?40111011011210101111011001?03??01??????????????????????????1011??????110010000?110101???????????????????111??01??01111111001101?10111?00?11101?11?1?1?11??0????????0000

Jobaria 11110011111100010101100001011101110?10?11?000??000?001000???????11101110000001?3011000100?310101111111000002110100100000001100100000000???10001?001011010100000101011110100101110000111111000101011111010011011101111001111011111????????0??????????00

Malawisaurus 11?????1??1???????????????????????????????????????????100???????1?1??11210?011??1100011?0??10101?01?1101110???????1??10?001110100001000???101?0?11??1?????00011101011000100???1??111????????????111?111?????11110???????1?????????????1??1???????0????

Nigersaurus 0011??1100???0011?001???1????1?1110???????0001?00??01110?100????22110212010101??0110001?0?????????????????????????????????????????????????1100???????1020?0???????????????????????????????????????????????????????????????????????????????0???????????

Rayososaurus ?????????01??0011?00110?1???110?110??0????100101011011????????????1??212???101??011000100??1?10???1110000?1?????0010000000110110000000011?1?????1???11020?000101000111101001????????????11??01010111110?00?10??1?1???0??10001111?????????0????????????

Rebbachisaurus ?????????????????????????????????????????????????????????????????????????????1??????????0??11??111111000011?????????????????????????????????????????110201?????1?0??????????????????????????????011??????????????????????????????????????0????????????

Alamosaurus ????????????????????????????????????????????????????????????????????????????11??1100001?0??1010110101101110??????1231100001101100001010???????0?111111101111?11111101100111???1111112??????????0111??????????????????????????????????????0????????????

Nemegtosaurus 01?1?011?10100010101010101010101111211111?11000100010110111??01?111?02121000??????????????????????????????????????????????????????????????????????????????????????????????????????????????????????????????????????????????????????????????01?10??01111

Neuquensaurus ????????????????????????????????????????????????????????????????????????????11??010?001?0??101????1?1?0111031?0?1??3010?0011?110000101110???????????11101011111111101101111?????????????111101?011111211111111?1011101??1110?????1???????1????????????

Opisthocoelicaudia ?????????????????????????????????????????????????????????????????????????????1?????????0104101011110110111031?0??1221200001101100001020100??110?1111110011111111111011011111??1111112??1111?0110111112111011111101110111111011111111011110????????????

Rapetosaurus 0011?111?10?000?000101010101010?1112111???11?0100?110100111?????11110212100011??110?011?0??10101?01011011103?????????1??0???????00???1????1?????????1110??10011101101?00110?????1?1????11101011011111?1101???????????????????????????????11?1110110?00

Saltasaurus ?????????????00??101?00101????????????????01000101???1??????????????????????11??011000110??10101?1101101110312011????10?00111110000101110?1?????11?011101011111111101101111????????????11111011011111211111111110????????????????????????1????????????

Isisaurus ?????????????????????????????????????????????????????????????????????????????1??110000100??101011010100?1103120??????10?001101100001010???1??10?11?0101010????1101111101???????????????111110110111???????????????????????????????????????????????????

Tapuiasaurus 00?1?111?10?100101?10??10101110111??1111??11?0?00?110?001?0??1??111102121?0011??01???11????10?0110101??011??????????????????????????????????11??????1?????10?1???1??110011??????1??????????????????1?2?????????1?????????????????1110?111?111111111111

Phuwiangosaurus ???????????????10101????0?????0?110???????100011010001????????????110212???001??0110011010?1010111101100100?1?0100?00000?0110010000000010011110?101?110111???10101011?011101???????????11101011111111211011101111????????111?????1???????0???????01?0?

Tangvayosaurus ?????????????????????????????????????????????????????????????????????????????1???1????????????01?11???0?10??????00?00000?0??11100000000100???10?111?1???????????????????????????????????????01101111121?0?110111011100???1001111?111001110????????????

Diamantinasaurus ????????????????????????????????????????????????????????????????????????????11??????????????????????????????????????????????????????????????11??????111010???11111111101???1??11?1111111111101101111121001111111111100????????????????????????????????

**References (Text S2)**

1. Wilson JA (2002) Sauropod dinosaur phylogeny: critique and cladistic analysis. Zool. J. Linn. Soc. 136: 217-276.
2. Curry Rogers KA (2005) Titanosauria: a phylogenetic overview. In: Curry Rogers KA, Wilson JA, Editors. The Sauropods: Evolution and Paleobiology. Berkeley: University of California Press. pp. 50-103.
3. Upchurch P, Barrett PM, Dodson P (2004) Sauropoda. In: Weischampel DB, Dodson P, Osmólska H, Editors. The Dinosauria. Edition 2. Berkeley: University of California Press. pp. 259-324.
4. Wilson JA, Upchurch P (2009) Redescription and reassessment of the phylogenetic affinities of *Euhelopus zdanskyi* (Dinosauria: Sauropoda) from the Early Cretaceous of China. J. Syst. Paleontol. 7: 199-239.
5. Suteethorn S, Le Loeuff J, Buffetaut E, Suteethorn V, Talubmook C et al. (2009) A new skeleton of *Phuwiangosaurus sirindhornae* (Dinosauria, Sauropoda) from NE Thailand. In: Buffetaut E, Cuny G, Le Loeuff J, Suteethorn V, Editors. Late Paleozoic and Mesozoic ecosystems in SE Asia. London: The Geological Society Special Publications 315. pp. 189-215.
6. Hocknull SA, White MA, Tischler TR, Cook AG, Calleja ND et al. (2009) New Mid-Cretaceous (Latest Albian) Dinosaurs from Winton, Queensland, Australia. PLoS ONE 4: 1-51.
