# Supplementary material for: A Complete Skull of an Early Cretaceous Sauropod and the Evolution of Advanced Titanosaurians
Source: PLoS One. 2011 Feb 7;6(2):e16663. doi: 10.1371/journal.pone.0016663 (PMC3034730; doi:10.1371/journal.pone.0016663)
Supplement: Text S3 — List of unambiguous synapomorphies. (DOC) [file pone.0016663.s009.doc]

**TEXT S3. LIST OF UNAMBIGUOUS SYNAPOMORPHIES.**

The nodes of the two most parsimonious trees are diagnosed by the following list of unambiguous synapomorphies. Node numbers refer to those shown in the strict consensus of Figure S4. Names have been added for nodes that represent broadly used clade names (otherwise the clade is referred given the most basal taxon name followed by “+MDS” [i.e., “and more derived sauropods”; *sensu* 1]):

Node 35 – Sauropoda:

Unambiguous synapomorphies present in both trees:

Char. 108: 0 → 1

Char. 127: 0 → 1

Char. 149: 0 → 1

Char. 160: 0 → 1

Char. 165: 0 → 1

Char. 167: 0 → 1

Char. 169: 0 → 1

Char. 172: 0 → 1

Char. 185: 0 → 1

Char. 192: 0 → 1

Char. 194: 0 → 1

Char. 196: 0 → 1

Char. 198: 0 → 1

Char. 211: 0 → 1

Char. 216: 0 → 1

Char. 222: 0 → 1

Char. 225: 0 → 1

Char. 230: 0 → 1

Char. 231: 0 → 1

Node 36 – (*Patagosaurus* + Barapasaurus + Mamenchisaurus + Omeisaurus + MDS):

Unambiguous synapomorphies present in both trees:

Char. 81: 1 → 0

Char. 94: 0 → 1

Char. 96: 0 → 1

Char. 99: 0 → 1

Char. 100: 0 → 1

Char. 101: 0 → 1

Char. 109: 0 → 1

Char. 150: 0 → 1

Unambiguous synapomorphies present only in one tree:

Char. 1: 0 → 1

Char. 18: 0 → 1

Char. 26: 0 → 1

Char. 78: 0 → 1

Char. 108: 1 → 2

Char. 112: 0 → 1

Char. 139: 0 → 1

Char. 182: 0 → 1

Char. 224: 0 → 1

Node 37 – Eusauropoda:

Unambiguous synapomorphies present in both trees:

Char. 190: 0 → 1

Char. 197: 0 → 1

Char. 204: 0 → 1

Char. 208: 0 → 1

Char. 217: 0 → 1

Char. 221: 0 → 1

Char. 223: 0 → 1

Char. 226: 0 → 1

Char. 227: 0 → 1

Char. 229: 0 → 1

Char. 232: 0 → 1

Char. 233: 0 → 1

Node 38 – (*Omeisaurus* + *Mamenchisaurus*):

Unambiguous synapomorphies present in both trees:

Char. 72: 0 → 1

Char. 86: 0 → 1

Char. 87: 1 → 0

Unambiguous synapomorphies present only in one tree:

Char. 80: 3 → 4

Node 39 – Diplodocidae:

Unambiguous synapomorphies present in both trees:

Char. 14: 0 → 1

Char. 15: 0 → 1

Char. 80: 3 → 4

Char. 88: 0 → 1

Char. 122: 0 → 1

Char. 129: 0 → 1

Char. 130: 0 → 1

Node 40 – Flagelicaudata:

Unambiguous synapomorphies present in both trees:

Char. 56: 0 → 1

Char. 85: 0 → 1

Char. 89: 0 → 1

Char. 95: 0 → 1

Char. 121: 0 → 1

Char. 128: 0 → 1

Char. 145: 1 → 0

Char. 189: 0 → 1

Char. 194: 1 → 0

Char. 195: 1 → 0

Char. 220: 0 → 1

Node 41 – (Rebbachisauridae + Flagelicaudata):

Unambiguous synapomorphies present in both trees:

Char. 102: 1 → 0

Char. 126: 0 → 1

Node 42 – Diplodocoidea:

Unambiguous synapomorphies present in both trees:

Char. 140: 0 → 1

Node 43 – Neosauropoda:

Unambiguous synapomorphies present in both trees:

Char. 24: 0 → 1

Char. 72: 0 → 2

Char. 145: 0 → 1

Char. 173: 0 → 1

Node 44 – (*Jobaria* + MDS):

Unambiguous synapomorphies present in both trees:

Char. 4: 0 → 1

Char. 102: 0 → 1

Char. 123: 0 → 1

Char. 124: 0 → 1

Char. 175: 0 → 1

Char. 176: 0 → 1

Char. 195: 0 → 1

Char. 210: 0 → 1

Unambiguous synapomorphies present only in one tree:

Char. 154: 0 → 1

Char. 184: 0 → 1

Char. 203: 0 → 1

Node 45 – (*Diplodocus* + *Barosaurus*):

Unambiguous synapomorphies present in both trees:

Char. 86: 0 → 1

Char. 118: 0 → 1

Char. 119: 0 → 1

Char. 120: 0 → 1

Char. 131: 0 → 1

Char. 132: 0 → 1

Char. 133: 0 → 1

Node 46 – Titanosauriformes:

Unambiguous synapomorphies present in both trees:

Char. 49: 0 → 1

Char. 76: 0 → 1

Char. 85: 0 → 1

Char. 140: 0 → 1

Char. 178: 0 → 1

Char. 179: 0 → 1

Char. 187: 0 → 1

Char. 198: 0 → 1

Node 47 – Macronaria:

Unambiguous synapomorphies present in both trees:

Char. 50: 0 → 1

Char. 77: 0 → 1

Char. 86: 0 → 1

Char. 141: 0 → 1

Char. 179: 0 → 1

Char. 180: 0 → 1

Char. 188: 0 → 1

Char. 199: 0 → 1

Node 48 – Dicraeosauridae:

Unambiguous synapomorphies present in both trees:

Char. 19: 0 → 1

Char. 23: 0 → 1

Char. 27: 0 → 1

Char. 45: 0 → 1

Char. 47: 0 → 1

Char. 49: 0 → 1

Node 49 – (*Euhelopus* + MDS):

Unambiguous synapomorphies present in both trees:

Char. 100: 1 → 0

Char. 108: 2 → 3

Char. 153: 0 → 1

Node 50 – Lithostrotia:

Unambiguous synapomorphies present in both trees:

Char. 98: 1 → 0

Char. 106: 0 → 1

Char. 118: 0 → 1

Char. 132: 0 → 1

Char. 205: 0 → 1

Char. 234: 0 → 1

Node 51 – (*Tangvayosaurus* + MDS):

Unambiguous synapomorphies present in both trees:

Char. 146: 0 → 1

Char. 192: 1 → 0

Node 52 – (*Phuwiangosaurus* + MDS):

Unambiguous synapomorphies present in both trees:

Char. 68: 0 → 1

Char. 193: 0 → 1

Char. 198: 1 → 2

Char. 202: 0 → 1

Node 53 – Rebbachisauridae:

Unambiguous synapomorphies present in both trees:

Char. 152: 0 → 2

Char. 162: 1 → 0

Node 54 – Opishtocoelicaudinae:

Unambiguous synapomorphies present in both trees:

Char. 117: 0 → 1

Char. 148: 0 → 1

Char. 154: 0 → 1

Char. 234: 1 → 0

Node 55 – Saltasauridae:

Unambiguous synapomorphies present in both trees:

Char. 164: 1 → 0

Char. 201: 0 → 1

Char. 214: 0 → 1

Node 56 – (*Diamantinasaurus* + MDS):

Unambiguous synapomorphies present in both trees:

Char. 161: 0 → 1

Node 57 – (*Isisaurus* + MDS):

Unambiguous synapomorphies present in both trees:

Char. 86: 1 → 0

Char. 168: 0 → 1

Char. 187: 0 → 1

Node 58 – “Advanced Titanosaurs” (Nemegtosauridae + Saltasauridae + *Isisaurus* + *Diamantinasaurus*):

Unambiguous synapomorphies present in both trees:

Char. 134: 0 → 1

Char. 155: 0 → 1

Char. 163: 0 → 1

Node 59 – Nemegtosauridae:

Unambiguous synapomorphies present in both trees:

Char. 22: 0 → 1

Node 60 – Saltasaurinae:

Unambiguous synapomorphies present in both trees:

Char. 81: 1 → 0

Char. 135: 0 → 1

Node 61 – (*Rapetosaurus* + *Tapuiasaurus*):

Unambiguous synapomorphies present in both trees:

Char. 2: 1 → 0

Char. 6: 0 → 1

Char. 48: 1 → 0

Char. 55: 1 → 0

Char. 235: 0 → 1

Char. 239: 0 → 1

Char. 242: 0 → 1

**Reference (Text S3)**

1. Wilson JA (2002) Sauropod dinosaur phylogeny: critique and cladistic analysis. Zool. J. Linn. Soc. 136: 217-276.
