# Supplementary material for: A Complete Skull of an Early Cretaceous Sauropod and the Evolution of Advanced Titanosaurians
Source: PLoS One. 2011 Feb 7;6(2):e16663. doi: 10.1371/journal.pone.0016663 (PMC3034730; doi:10.1371/journal.pone.0016663)
Supplement: Text S4 — Age of first appearance for taxa used in the calibrated phylogeny. (DOC) [file pone.0016663.s010.doc]

**TEXT S4. AGE OF FIRST APPEARANCE FOR TAXA USED IN THE CALIBRATED PHYLOGENY DEPICTED IN FIGURE 3.**

| **Taxa** | **Age** | **Reference** |
| --- | --- | --- |
| *Barosaurus* | Kimmeridgian-Tithonian | Upchurch *et al.* (2004) |
| *Diplodocus* | Kimmeridgian-Tithonian | Upchurch *et al.* (2004) |
| *Apatosaurus* | Kimmeridgian-Tithonian | Upchurch *et al.* (2004) |
| *Dicraeosaurus* | Kimmeridgian | Upchurch *et al.* (2004) |
| *Amargasaurus* | Barremian | Leanza *et al.* (2004) |
| *Haplocanthosaurus* | Kimmeridgian-Tithonian | Upchurch *et al.* (2004) |
| *Nigersaurus* | Aptian-Albian | Sereno and Wilson (2005) |
| *Rayososaurus* | Cenomanian | Carballido *et al.* (in press) |
| *Rebbachisaurus* | Cenomanian | Upchurch *et al.* (2004) |
| *Camarasaurus* | Kimmeridgian-Tithonian | Upchurch *et al.* (2004) |
| *Brachiosaurus* | Kimmeridgian-Tithonian | Upchurch *et al.* (2004) |
| *Euhelopus* | Baresian-Albian | Wilson and Upchurch (2009) |
| *Phuwiangosaurus* | Barremian-Aptian | Racey and Goodall (2009) |
| *Tangvayosaurus* | Aptian-Albian | Upchurch *et al.* (2004) |
| *Malawisaurus* | Aptian | Upchurch *et al.* (2004) |
| *Nemegtosaurus* | Early Maastrichtian | Upchurch *et al.* (2004) |
| *Tapuiasarus* | Aptian | This study |
| *Rapetosaurus* | Campanian-Maastrichtian | Rogers *et al.* (2000) |
| *Isisaurus* | Middle-Late Maastrichtian | Upchurch *et al.* (2004) |
| *Diamantinasaurus* | Latest Albian | Hocknull *et al.* (2009) |
| *Saltasaurus* | Late Campanian-Maastrichtian | Upchurch *et al.* (2004) |
| *Neuquensaurus* | Early Campanian | Salgado *et al.* (2005) |
| *Opisthocoelicaudia* | Early Maastrichtian | Upchurch *et al.* (2004) |
| *Alamosaurus* | Maastrichtian | Upchurch *et al.* (2004) |

**References (Text S4)**

J. L. Carballido, C. A. Garrido, I. Canudo, L. Salgado, Redescription of *Rayososaurus agrioensis*. *Geobios* (in press)*.*

21. Hocknull SA, White MA, Tischler TR, Cook AG, Calleja ND et al. (2009) New Mid-Cretaceous (Latest Albian) Dinosaurs from Winton, Queensland, Australia. PLoS ONE 4: 1-51.

H. A. Leanza, S. Apesteguía, F. E. Novas, M. de la Fuente, Cretaceous beds from the Heuquén basin (Argentina) and their tetrapod assemblages. *Cretaceous Research* **25**, 61-87 (2004).

36. A. Racey, J. G. S.Goodall, in *Late Palaeozoic and Mesozoic Ecosystems in SE Asia*, E. Buffetaut, G. Cuny, J. Le Loeuff, V. Suteethorn, Eds. (Geological Society Special Publications 315, London, 2009), pp. 67-81.

37. R. R. Rogers, J. H. Hartman, D. W. Krause, Stratigraphic analysis of Upper Cretaceous rocks in the Mahajanga Basin, northwestern Madagascar: implications for ancient and modern faunas. *Journal of Geology* **108**, 275–301 (2000).

38. L. Salgado, S. Apesteguía, S. E. Heredia, A new specimen of *Neuquensaurus australis*, a Late Cretaceous saltasaurinae titanosaur from North Patagonia. *J. Vert. Paleontol.* **25**, 623-634 (2005).

P. C. Sereno, J. A. Wilson, in *The Sauropods: Evolution and Paleobiology*, K. A. Curry Rogers, J. A. Wilson, Eds. (University of California Press, Berkeley, 2005), pp. 157-177.

P. Upchurch, P. M. Barrett, P. Dodson, in *The Dinosauria*, D. B. Weischampel, P. Dodson, H. Osmólska, Eds. (University of California Press, Berkeley, ed. 2, 2004). 259-324

32. J. A. Wilson, P. Upchurch, Redescription and reassessment of the phylogenetic affinities of *Euhelopus zdanskyi* (Dinosauria: Sauropoda) from the Early Cretaceous of China. *J. Syst. Paleontol.* **7**, 199-239 (2009).
